# Origin and dispersal of Hepatitis E virus

**DOI:** 10.1038/s41426-017-0009-6

**Published:** 2018-02-07

**Authors:** Diego Forni, Rachele Cagliani, Mario Clerici, Manuela Sironi

**Affiliations:** 1 Scientific Institute IRCCS E. MEDEA, Bosisio Parini, 23842 Italy; 2 Department of Pathophysiology and Transplantation, University of Milan, Milan, 20090 Italy; 3 Don C. Gnocchi Foundation ONLUS, IRCCS, Milan, 20148 Italy

## Abstract

Hepatitis E virus (HEV, genus *Orthohepevirus*) is a common cause of hepatitis worldwide. Human-infecting HEV strains (*Orthohepevirus A*) include human-restricted and enzootic genotypes. Viruses in the *Orthohepevirus A* species also infect rabbits (HEV-3ra), camels, and swine. Using a selection-informed method, we dated the origin of the *Orthohepevirus* genus at least 21 million years ago, whereas the *Orthohepevirus A* species originated in Asia, most likely from a human-infecting ancestor that existed ~4500 to 6800 years ago. In this period, the appearance of large human settlements probably facilitated HEV emergence and spread. The earliest events in *Orthohepevirus A* evolutionary history involved the separation of the enzootic and human-restricted genotypes, as well as the split of the camel-infecting genotypes, which occurred during the time-frame of camel domestication. The place and timing of HEV-3ra divergence also correspond to the circumstances of rabbit domestication. This study clarifies the origin and historical events underlying HEV dispersal.

## Introduction

Hepatitis E virus (HEV) is the most common cause of enterically-transmitted viral hepatitis worldwide^1^. HEV infection usually causes acute self-limiting hepatitis, but fulminant hepatic failure can occur in pregnant women, elderly patients or individuals suffering from underlying chronic liver disease^1^. In immunocompromised patients, HEV infection can develop into chronic hepatitis^1^. Overall, HEV imposes a significant health burden, with 20 million estimated annual infections, 3.3 million symptomatic cases, and more than 50,000 HEV-related deaths (http://www.who.int/mediacentre/factsheets/fs280/en/).

HEV is a single stranded, positive RNA virus belonging to the *Hepeviridae* family. Members of this family are classified into two genera, *Orthohepevirus* and *Piscihepevirus*^2^. The Piscihepevirus genus includes only one species with one member (cutthroat trout virus), whereas the *Orthohepevirus* genus is divided into four species (Orthohepevirus A to D)^2^. Human-infecting HEV strains belong to the *Orthohepevirus A* species: genotypes 1 and 2 (HEV-1 and HEV-2) infect only humans, whereas genotypes 3 and 4 (HEV-3 and HEV-4) have been described in humans and other domestic (mainly pigs) and wild animals^1^. Additional Orthohepevirus A genotypes were detected in rabbits (HEV-3ra), boars (HEV-5 and HEV-6), and camels (HEV-7 and HEV-8)^1^. Other species in the *Orthohepevirus* genus infect birds (*Orthohepevirus B*), rats and ferrets (Orthohepevirus C), as well as bats (Orthohepevirus D)^1^. Additional HEV sequences identified in mammals and birds are distantly related to other HEV species and remain unclassified^1^.

Human-infecting HEV genotypes display distinct epidemiological patterns: HEV-1 and HEV-2 cause waterborne outbreaks mainly in tropical and subtropical regions^1^, whereas the zoonotic transmission of HEV-3 and HEV-4 accounts for the majority of hepatitis E human cases in industrialized countries^1^. Phylogenetic analyses of HEV-3 and HEV-4 sequences from humans and swine revealed no clustering by host species^3^. This observation, and the ability of viruses derived from swine to infect non-human primates^1^, suggest that pig-infecting HEV-3 and HEV-4 can readily cross the species barrier and infect humans. Likewise, cynomolgus macaques can be infected with rabbit HEV, and a human HEV strain closely related to HEV-3ra was isolated, suggesting inter-species transmission of the virus from rabbits to humans^4, 5^. Recently, the identification of HEV-7 in a patient who regularly consumed camel meat and milk suggested that this genotype is also able to infect our species^6^. To summarize, whereas it seems that all *Orthohepeviruses A* are transmissible to humans, experimental infection with human-derived HEV-1 and HEV-2 strains indicated that these viruses have a limited host range, which is virtually limited to primates^1^. HEV genotypes are therefore usually referred to as enzootic (HEV-3 and 4) or human-restricted/anthropotropic (HEV-1 and 2).

The evolutionary events that led to the origin and radiation of the major* Orthohepevirus* species, as well of the *Orthohepevirus A* genotypes remain poorly understood. An analysis conducted before the identification of the boar and camel-derived genotypes indicated that the ancestor of HEV genotypes 1–4 split into human-restricted and enzootic lineages in relatively recent times, about 536 to 1344 ya^7^. The same study suggested that the ancestor of all Orthohepevirus A was enzootic^7^.

Herein we used a large set of HEV sequences to investigate the geographic origin and ancestral host range of *Orthohepevirus A*, as well as to estimate the timing of speciation and genotype radiation.

## Materials and methods

### Geographic distribution of HEV genotypes

**Fig. 1:**
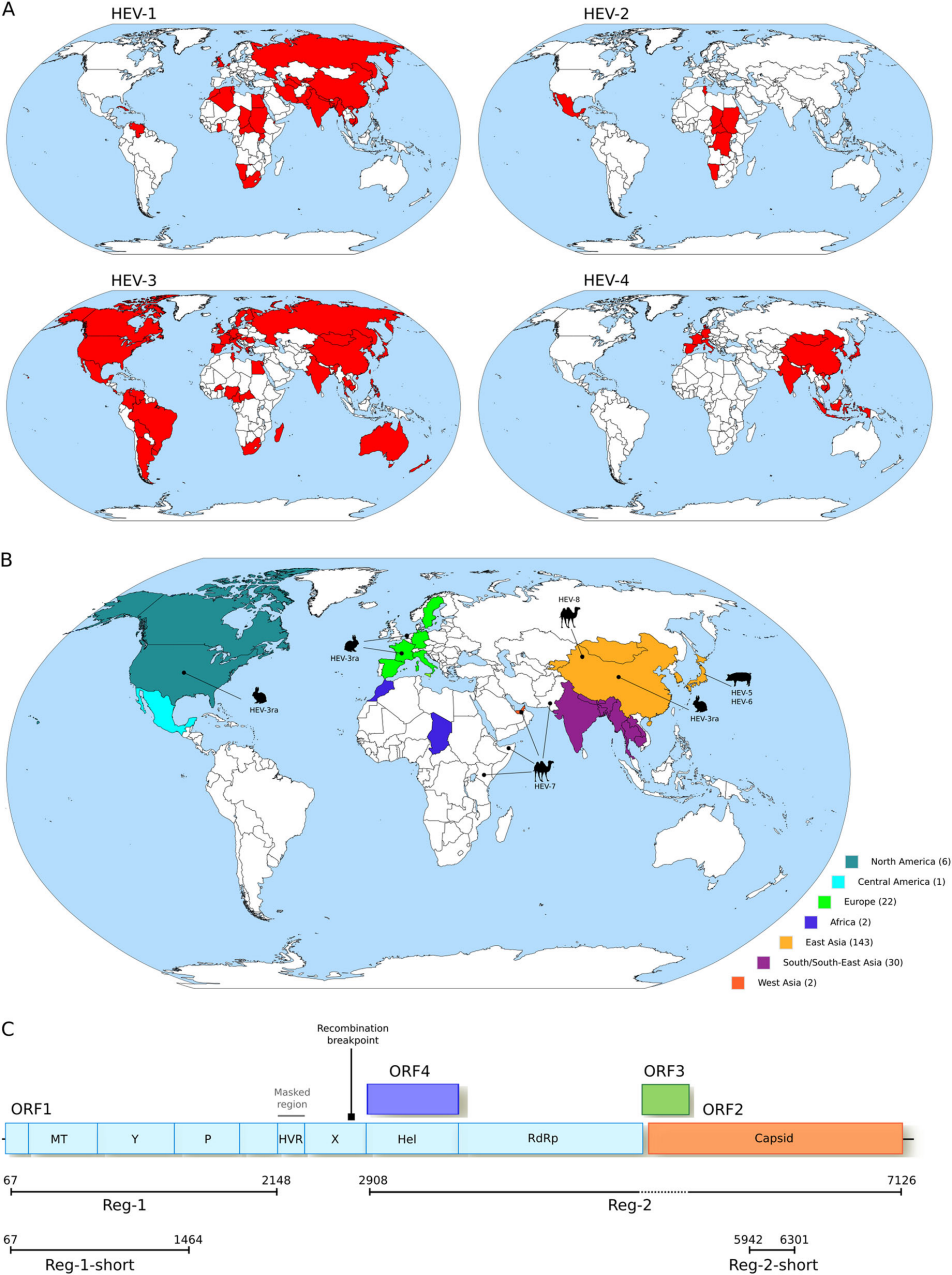
Worldwide distribution of HEV genotypes. **a** Geographic distribution of the four major HEV genotypes (HEV-1 to HEV-4). **b** Geographic origin of complete *Orthohepevirus A* genomes analyzed in this study. The number of sequences deriving from each continent or region is reported in parentheses. The location where animal-infecting *Orthohepevirus A* were collected is also shown. **c** Schematic representation of the *Orthohepevirus A* genome. Positions refer to the Burma (GenBank accession: M73218) reference strain for genotype 1. Note that ORF4 has been described only in HEV-1 and not in other HEV genotypes^25^. The regions we used in different analyses, as detailed in the text, are shown. The region we filtered from the *Orthohepevirus A* genome alignment is indicated. The location of the recombination breakpoint is also reported. *MT* methyltransferase, *P* papain-like cysteine protease, *Hel* helicase, *RdRp* RNA-dependent RNA polymerase, *Y* Y domain, *HVR* hypervariable region, also known as polyproline region (PPR); X, macro domain

To obtain an overview of the geographic distribution of *Orthohepevirus A* genotypes, we updated epidemiological surveys^8–14^ (Supplementary Table S1) with recent literature reports of molecular-typed HEV infections, as well as with information on HEV strains identified in animals (Figs. 1a, b). Genotypes were assigned to countries irrespective of their prevalence. Thus, even if a single case was reported in a given country, the genotype was recorded as present. Cases that could be clearly ascribed to migration/travels were excluded.

### Sequences, alignments, and recombination

All complete or almost complete *Orthohepevirus* genomes were retrieved from the ViPR database (https://www.viprbrc.org/)^15^. As of 1 March 2017, 289 *Orthohepevirus A* sequences were available. Information on collection date and host, as well as geographic origin, were retrieved either via ViPR or through manual inspection of the literature. We discarded sequences with incomplete information. HEV strains deriving from cell culture adaptation, recombinants, and sequences isolated from experimentally inoculated animals were also pruned. The final dataset comprised 206 *Orthohepevirus A* sequences and included most strains proposed as references for HEV genotypes (with the exclusion of reference strains for genotypes 2b and 3d, as only partial sequences are available) (Supplementary Table S2). As for *Orthohepevirus B* to *D*, a total of 31 complete genomes were available, all of them with complete information about sampling date and location, as well as host (Supplementary Table S2). The sequences of three unclassified Orthohepeviruses deriving from a Swedish moose, a little egret, and a common kestrel^16–18^ were also downloaded from ViPR, as well as the genome sequence of cutthroat trout virus^19^ (Supplementary Table S2).

To root phylogenies, the outgroup method was used. In particular, the viral sequence falling outside the ingroup and showing the greatest phylogenetic proximity was selected^20^. Thus, the cutthroat trout virus^19^, which is the only member of the Piscihepevirus genus, was selected as the outgroup to root the *Orthohepevirus* phylogeny. As for analyses restricted to *Orthohepeviruses* *A*, the moose-derived virus was used for rooting. In fact, this virus falls outside the *Orthohepevirus* *A* species, but is more closely related to *Orthohepeviruses* *A* than other *Orthohepevirus* species (B to D)^16^.

A whole genome alignment of *Orthohepevirus* *A* sequences was generated using MAFFT^21^. The region corresponding to the hypervariable region (HVR) was filtered due to very poor alignment quality and recombination was searched for using the genetic algorithm GARD^22^ implemented in the HYPHY package^23^. GARD uses phylogenetic incongruence among segments in an alignment to detect the best-fit number and location of recombination breakpoints and evaluates the statistical significance of putative breakpoints through Kishino-Hasegawa tests. Because we detected one statistically significant (*P* < 0.01) breakpoint, two subregions, Region-1 (Reg-1) and Region-2 (Reg-2) were used in all analyses. In particular, Reg-1 includes the coding portion showing good alignment quality located 5′ of the recombination breakpoint (Fig. 1c). Reg-2 covers the helicase and RdRp domains in ORF1 plus the ORF2 region that shows no overlap with ORF3 (Fig. 1c). To increase the alignment length and, consequently, the confidence in phylogenetic reconstruction^24^, the helicase domain was included even though an alternative reading frame ORF (ORF4) was described (Fig. 1c); however ORF4 was only reported for HEV-1^25^. The ORF1 and ORF2 segments were concatenated.

For phylogeographic analysis, we also used a sub-region of Reg-2 (denoted Reg-2-short). Reg-2-short was selected to maximize the number of available African and South/Central American sequences. In particular, we downloaded from ViPR all HEV genome fragments longer than 300 nucleotides and deriving from sequences isolated in Africa and South/Central America. We aligned these fragments to complete HEV genomes and selected the region of highest coverage with a minimum alignment length of 300 bp. This region corresponded to Reg-2-short, with an alignment length of 360 nucleotides.

Alignments of all regions were generated using RevTrans 2.0^26^, which uses the protein alignment as a scaffold to build the nucleotide alignment.

### Phylogeographic analysis and reconstruction of ancestral host range

For the phylogeographic analysis of complete HEV genomes, sequences were assigned to continents. As the majority of complete genomes came from Asia, this continent was divided into sub-region, namely East Asia, West Asia, and South/South-East Asia, based on the United Nation geographical sub-regions (http://unstats.un.org/unsd/methods/m49/m49regin.htm). Areas and the number of sequences per area are shown in Fig. 1b.

As for the phylogeographic analysis of Reg-2-short, 48 African sequences were available. We thus selected 48 Reg-2-short sequences from those sampled in South/Central America (out of a total of 61) and the same number from Asian, European, and North American sequences. All these sequences were selected to be distinct from those used in the full genome analyses and to be representative of collection date and country. Because only 31 South/South-East Asian sequences were available (and fewer for West Asia), East, West, and South/South-East Asia were collapsed in a single area (Asia) (Supplementary Table S3).

Inference of evolutionary rates, geographical origin, and ancestral host range were obtained using the Bayesian Evolutionary Analysis by Sampling Trees (BEAST, version 2.4.4) software^27^. Analyses were performed using the Bayesian Markov Chain Monte Carlo (MCMC) method with a General Time Reversible (GTR) substitution model and a gamma distribution (G) rate with 4 categories among sites. The GTR + G model was selected using the “ModelTest” utility^28^ implemented in the HYPHY package. A strict molecular clock was used.

The geographical locations and ancestral hosts at internal nodes were estimated using the discrete model implemented in BEAST^29^. Two different runs, one hundred million iterations each, were performed and sampled every 10 000 steps with a 10% burn-in. Runs were then combined after checking for convergence. Maximum clade credibility trees were summarized using TreeAnnotator^27^. All analyzed nodes had a posterior probability higher than 0.99. Trees were visualized with FigTree (http://tree.bio.ed.ac.uk/).

Both ancestral characters (geographic origin and host range) were also inferred using the BBM (Bayesian Binary MCMC) method implemented in RASP (Reconstruct Ancestral State in Phylogenies)^30, 31^. For BBM analyses, 10 000 BEAST-generated trees and consensus tree were used as topology input. Two BBM chains were run for 100 000 generation with estimated state frequencies (F81), a gamma distributed among-site rate variation, sampling every 100 generations, and null character state for the outgroup (Swedish moose-derived *Orthohepevirus*).

BBM was preferred over other methods in the RASP suite for several reasons: (1) DEC and S-DEC (Statistical Dispersal–Extinction–Cladogenesis) could not handle the large number of nodes; (2) S-DIVA (Statistical-Dispersal Vicariance Analysis) favors models of vicariance and ancestral areas can be wrongly identified when evolutionary patterns are more complex^30, 32^; 3) BBM allows null character status information for a portion of input sequences. This property was exploited to run 100 analyses to check for consistency against the skewed geographic origin and host species of available sequences. In particular, for ancestral host range, most viruses were isolated from humans (*n* = 108) and swine (*n* = 60); we thus generated 100 distributions by randomly omitting character state for 22 human-derived sequences sampled from genotypes 3 and 4. For each distribution, we recorded the two highest probabilities, which always corresponded to human and swine.

For phylogeographic analysis of complete HEV genomes, 100 distributions were generated for BBM analysis so as to include a similar number of sequences from East-Asia, South/South-East Asia, and Europe. For each of these distribution, location probabilities were recorded for the MRCA and selected internal nodes. Probabilities were averaged across the 100 runs and the result displayed as pie charts.

### Time estimates

The action of purifying selection can bias tMRCA evaluation, and selection-informed models can improve branch length estimation^33, 34^. We thus applied a branch-site model (aBS-REL, adaptive branch-site random effects likelihood)^35^ to estimate branch lengths while taking into account the effect of different selective pressures among sites and lineages in the phylogeny.

To determine whether there was sufficient temporal structure in the HEV phylogenies to estimate divergence times, we used TEMPEST to perform a regression of root-to-tip genetic distances against year of sampling^36^. Evidence for temporal structure was obtained for both Reg-1 (*R*^2^ = 0.26) and Reg-2 (*R*^2^ = 0.24).

Estimates of divergence times were performed with the LSD (least-squares dating) software v0.2^37^. The aBS-REL tree was used as the input tree and in the case of extremely long terminal branches, most likely resulting from low precision in point estimates of dN/dS, branch lengths calculated using the GTR + G model were used instead of aBS-REL lengths.

A latin hypercube sampling scheme was used to sample from the aBS-REL parameter distributions so as to estimate confidence intervals, as previously suggested^33, 34^. Briefly, 500 samples were drawn from aBS-REL analyses to estimate branch length variance, 500 trees were generated, and then used as input trees for LSD. The upper and the lower 95% bounds were used as confidence intervals.

As a comparison, timescale trees were also estimated using the MCMC algorithm implemented in the BEAST package in combination with the GTR + G substitution model; we ran 2 chains for 100 million states with a step of 10 000. After discarding a 10 % burn-in and merging the two runs, the output was analyzed using TreeAnnotator.

tMRCA estimates of the origin of the *Orthohepevirus* genus were obtained using a phylogeny that included all available *Orthohepevirus* complete sequences that are not classified as *Orthohepevirus A* plus a sub-sample of *Orthohepevirus A* (*n* = 82, Supplementary Table S2), which were selected to be representative of different collection dates, geographic origins and hosts. Branch lengths were estimated using phyML with a maximum-likelihood approach, a General Time Reversible (GTR) model plus gamma-distributed rates, and four substitution rate categories^38^. Substitution rates and the dates of ancestral nodes were estimated using LSD. Branch lengths were also obtained using aBS-REL to account for the effect of variation in selective pressure along branches and to re-estimate ancestral node dating. The cutthroat trout virus sequence was used to root the phylogeny.

## Results

### Worldwide distribution of HEV genotypes and phylogeographic reconstruction

To obtain an overview of the geographic distribution of *Orthohepevirus A* genotypes, we combined data from human infections with information on HEV strains identified in animals (Supplementary Table S1). Results were consistent with known patterns^1^: HEV-1 and HEV-2 mainly occur in tropical and subtropical regions of Asia, Africa, and Latin America; HEV-4 is mostly observed in Asia and Europe; and HEV-3 is distributed worldwide (Fig. 1a). Inclusion of geographic information for HEV-3ra, HEV-5/6, and HEV-7/8 indicated that East Asia hosts the largest diversity of HEV genotypes (Fig. 1b). Although this distribution may reflect recent events or more intense sampling in Asia, it is consistent with an East Asian origin of *Orthohepevirus A*. We thus decided to formally test this hypothesis using phylogeographic analyses.

We compiled a list of 206 *Orthohepevirus A* complete or almost complete genomes with information concerning date and place of collection, as well as host species (Supplementary Table S2). A genome alignment was generated and the HVR in ORF1 was filtered due to very poor alignment reliability. The resulting alignment was screened for the presence of recombination events using GARD^22^ and one breakpoint was detected immediately upstream the helicase domain of ORF1 (Fig. 1c). The location of this breakpoint corresponds to those previously identified using other methods and HEV sequence sets^39, 40^, suggesting that the two regions defined by the breakpoint have distinct evolutionary histories. Subsequent analyses were thus carried out on two regions: one that covers the 5′ region of ORF1 (Reg-1) and the other that comprises the helicase and RdRp domains of ORF1 plus ORF2 with the exclusion of the region of overlap with ORF3 (Reg-2) (Fig. 1c).

**Fig. 2:**
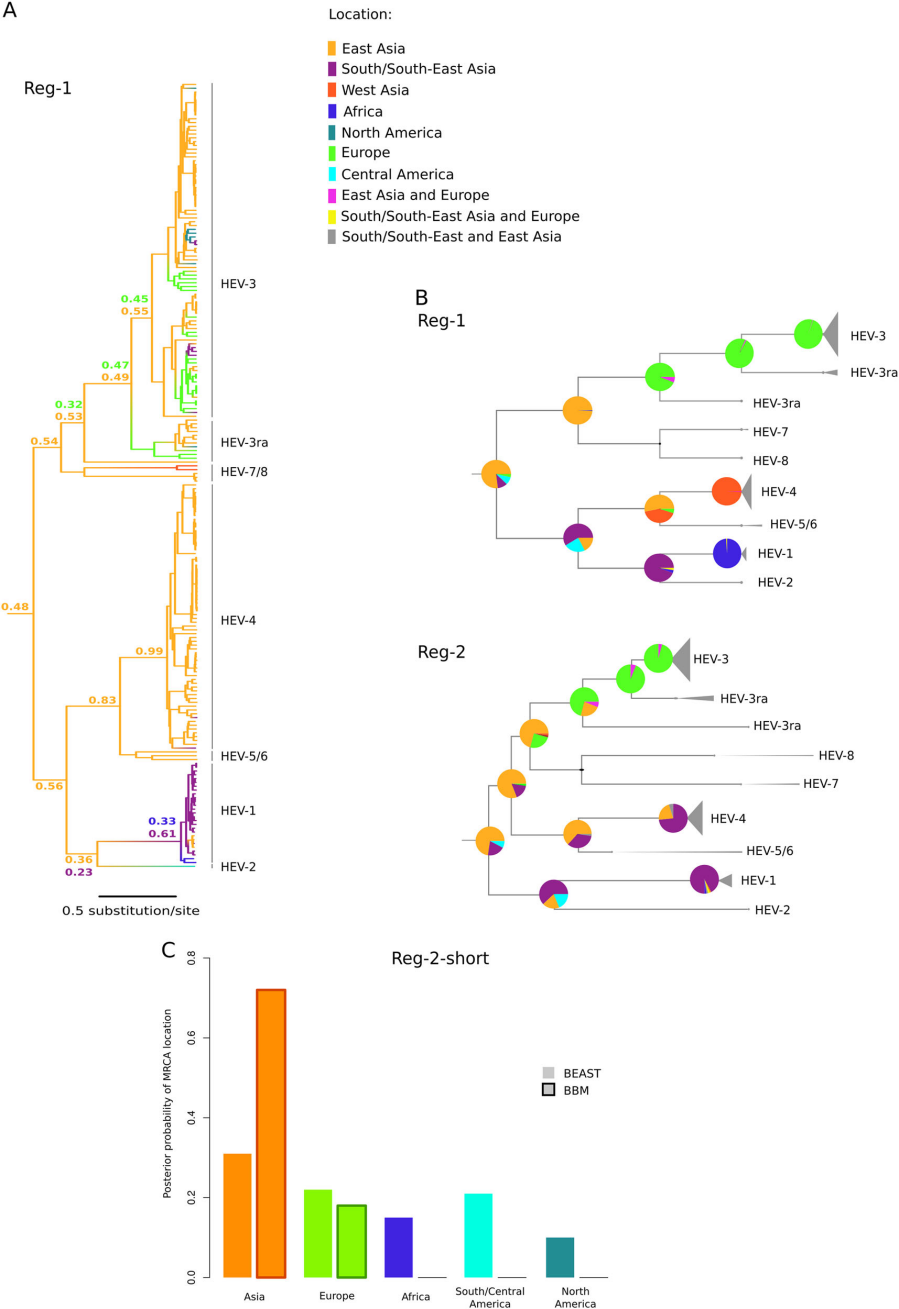
Phylogeographic analysis of *Orthohepevirus A*. **a** Maximum clade credibility tree for Reg-1. Branches are colored according to inferred ancestral location; posterior support for inferred locations at relevant nodes is shown. **b** The topologies of the Reg-1 and Reg-2 trees are shown. Pie charts represent the ancestral location probability of the respective area. Probabilities were averaged over 100 BBM runs with a similar number of sequences from East Asia, South/South-East Asia, and Europe. In BBM analyses, combined regions appear because we allowed two areas per node. **c** Posterior probability distributions for the location of the *Orthohepevirus A* MRCA obtained using BEAST and BBM. Results refer to the Reg-2-short region

We used two independent phylogeography methods to determine the likely location of *Orthohepevirus A* origin. Sequences were assigned to geographic areas (Fig. 1b), which represent the character states for ancestral state reconstruction. The discrete phylogeography analysis in BEAST^27, 29^ assigned the highest probability to East Asia as the origin of *Orthohepevirus A*, with posterior probabilities of 0.48 for Reg-1 and 0.53 for Reg-2. BEAST also inferred a likely East Asian origin for several internal nodes of the phylogeny (Fig. 2a, Supplementary Figure S1). The ancestors of extant HEV-3 strains, as well as the MRCA (most recent common ancestor) of HEV-3ra and HEV-3, were assigned roughly equal probabilities of European or East Asian origin (Fig. 2a, Supplementary Figure S1). Very similar results were obtained with the BBM method^31^, which assigned high probabilities of an East Asian origin to the ancestral node of the *Orthohepevirus A* phylogeny (0.93 for Reg-1 and 0.90 for Reg-2) (full tree not shown).

Most *Orthohepevirus A* sequences were collected in East Asia (mainly in China and Japan) (Fig. 1b). We thus checked whether this skewed distribution affected phylogeographic inference. To this aim, we run 100 BBM analyses by omitting the character state for 113 randomly selected East Asian strains. This number was selected to obtain a data set that included a similar number of sequences from East Asia (*n* = 30), South/South-East Asia (*n* = 30), and Europe (*n* = 22). We calculated the mean probabilities at selected nodes by averaging over the 100 BBM runs (Fig. 2b). For both ORFs, East Asia was identified as the most likely origin of *Orthohepevirus* *A*, with high average probabilities. These analyses also indicated that HEV-3 originated in Europe, in agreement with previous studies^41, 42^, and supported the notion that HEV-3ra is also of European origin. The MRCA of HEV-1 and HEV-2 were inferred to have originated in South/South-East Asia (Fig. 2b). This finding should however be interpreted with caution due to the availability of a single HEV-2 sequence.

The dataset of complete *Orthohepevirus A* genomes includes only few samples from Africa and South/Central America. To further confirm the Asian origin of *Orthohepevirus A*, we performed an additional analysis on a sub-region of Reg-2 (Reg-2-short, 360 nucleotide long) for which African and South/Central American sequences are available. The dataset for Reg-2-short included the same number of sequences (*n* = 48) for Asia, Africa, Europe, South/Central America, and North America (total sequences: 240) (see Methods and Supplementary Table S3). A drawback of this approach is that phylogenetic relationships can be reconstructed with limited confidence when small regions are analyzed^24^. Thus, we limited inference to the MRCA of the whole phylogeny, with no attempt to reconstruct the ancestral location of internal nodes. Both BEAST and BBM analyses inferred an Asian origin for the *Orthohepevirus A* MRCA (Fig. 2c). The second most likely location was Europe, with definitely lower probability, especially in BBM analysis (Fig. 2c). Overall, these data strongly support the hypothesis that *Orthohepevirus A* originated in Asia.

### Ancestral host of *Orthohepevirus A*

**Fig. 3:**
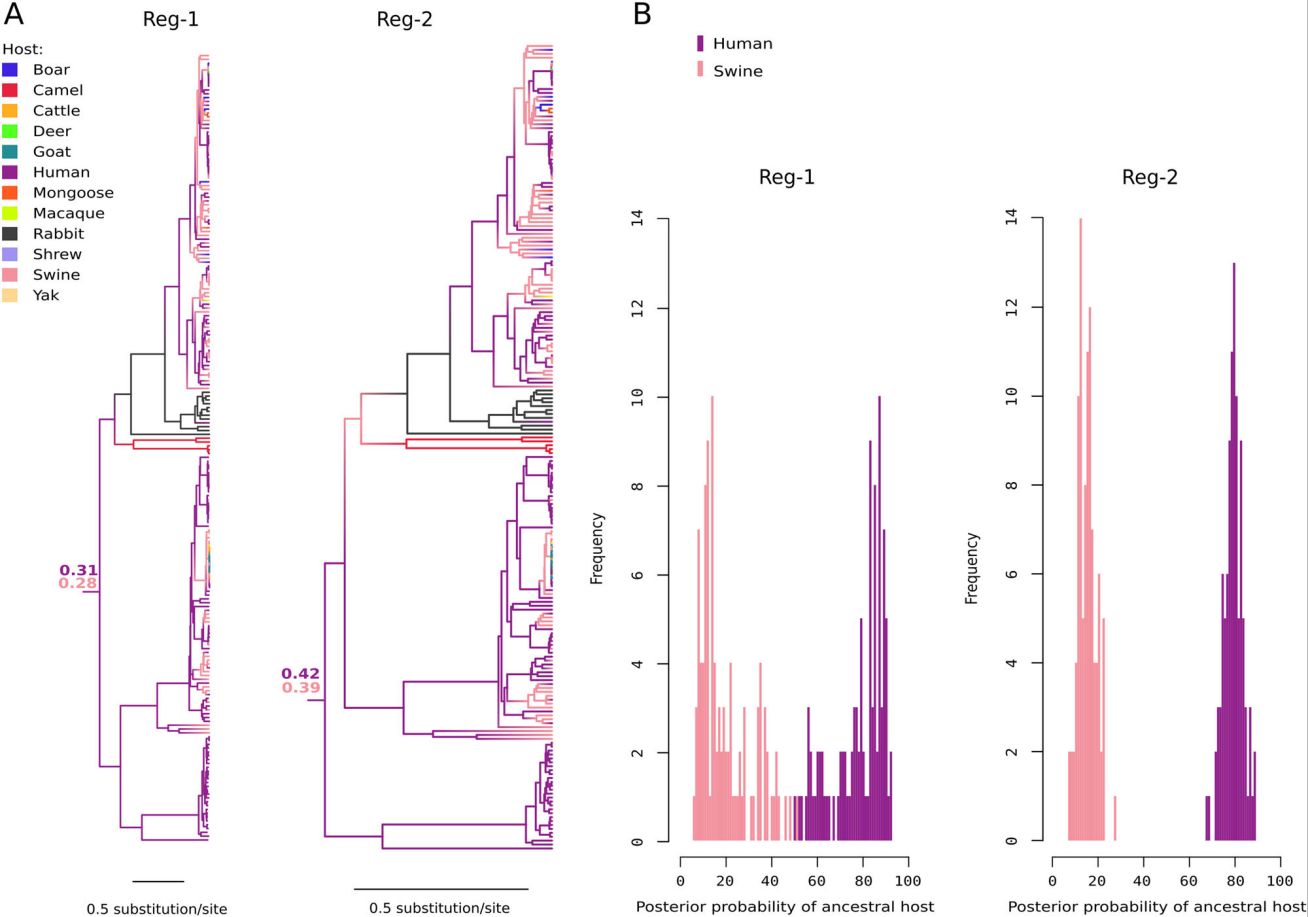
Ancestral host range of *Orthohepevirus A*. **a** Maximum clade credibility trees for Reg-1 and Reg-2. Branches are colored according to inferred ancestral host; posterior support for the host range of the MRCA is reported. **b** Posterior probability distributions of ancestral host state for the MRCA of the *Orthohepevirus A* phylogeny. Probabilities derive from 100 BBM runs with omission of character state information for 22 randomly sampled human sequences belonging to genotypes 3 and 4

As mentioned above, the major HEV genotypes differ in their host range, although most of them seem to be able to infect humans. Thus, an interesting question is whether the ancestor of extant *Orthohepeviruses A* infected humans or other mammals. We investigated this issue by applying an approach similar to the one described above. In this case, host information was entered as character state in BEAST and BBM analyses. BEAST indicated that the most likely ancestral host of *Orthohepevirus A* was human, although posterior probabilities were only slightly higher than those for swine (Fig. 3a). Similar results were obtained with BBM, with definitely higher probabilities of 0.94 and 0.86 for a human host for both regions (full tree not shown).

However, as is the case for geographic location, the source of HEV sequences is biased, with an over representation of human-derived viruses. We thus generated 100 character state distributions by omitting the information for 22 randomly selected human-derived strains belonging to genotypes 3 and 4. We did not omit character states for genotype 1 and 2, as these viruses exclusively infect humans (thus the ancestor of HEV-1 and HEV-2 is always inferred with high confidence to be human-derived). The resulting distributions, used as an input for BBM, have the same number of HEV-3 and HEV-4 sequences derived from humans and swine. For both regions, BBM always estimated the probability of a human ancestral host to be definitely higher than that of an ancestral swine host (Fig. 3b). For both regions, the distribution of probabilities was definitely skewed towards a human ancestral host (Fig. 3b).

### Dating the origin of *Orthohepevirus A*

Evolutionary rates scale negatively with the time-frame of the measurement for different viral taxa^43, 44^. This phenomenon often results in underestimation of the age of viral lineages^43, 44^ and is strongly associated with purifying selection and substitution saturation^43^. We thus applied a selection-informed method to estimate the tMRCA (time to the most recent common ancestor) of *Orthohepeviruses A*. Specifically, we calculated branch lengths in *Orthohepevirus A* phylogenies using the aBS-REL method, which allows for site- and branch-specific variation in selective pressure and is more robust than other evolutionary models to substitution saturation^34, 45^. These lengths were converted into tMRCA estimates using LSD (least-squares dating)^37^. Confidence intervals were calculated by estimating the variance in branch lengths produced by the aBS-REL model using a Latin hypercube sampling importance resampling scheme.

As above, we analyzed two regions, Reg-1 and Reg-2. Saturation of synonymous substitution rates (dS) was not prominent in the two regions, as we found 3.6 and 4.3% of branches (most of them terminal branches) showing dS saturation for Reg-1 and Reg-2, respectively. We estimated the tMRCA of* Orthohepevirus A* genotypes to be 6795 years ago (ya) for Reg-1 (95% IC:13871–4011) and 4596 ya for Reg-2 (95% IC:13806–2404). In line with previous results^45^, much shallower tMRCAs were obtained using the GTR tree lengths for LSD (Table 1) or BEAST (for Reg-1, tMRCA = 1484 ya, CI: 2049–1132; for Reg-2, tMRCA = 975 ya, CI: 1383–242) analyses.

**Table 1:** Branch lengths and tMRCA estimates for the *Orthohepevirus* genus

	Length (substitution/site)		Expansion (CI)^a^	tMRCA (ya)	
***Orthohepevirus***					
	**GTR**	**aBS-REL (CI)**		**GTR**	**aBS-REL** ^**b**^ **(CI)**
Reg-1-short	45.99	91 009	1978.89	24 812	49 100 219
		(40 200–103 923)	(874.10–2 259.69)		(21 688 169–56 067 428)
Reg-2	36.54	168 856	4621.13	7300	33 734 249
		(122 782–380 992)	(3 360.21–10 426.72)		(24 529 533–74 115 056)
***Orthohepevirus A***					
	**GTR**	**aBS-REL**		**GTR**	**aBS-REL** ^b^ **(LSD calculated)** ^**c**^
Reg-1	14.22	60.13	4.23	1884	7969 (6795)
Reg-2	12.19	86.72	7.11	878	6242 (4596)

^a^ Total branch length expansion (aBS-REL/GTR + G)

^b^ tMRCA inferred by multipling the time obtained with the GTR + G model (and LSD) by branch length expansion

^c^ tMRCA estimated using aBS-REL branch lengths as an input for LSD

**Fig. 4:**
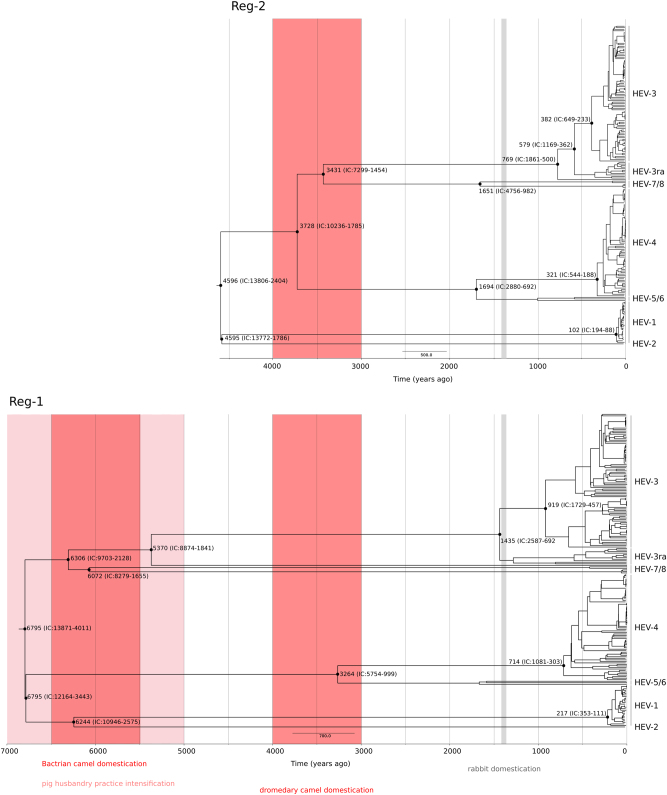
*Orthohepevirus A* timescaled phylogenetic trees. Timescaled phylogenetic tree estimated using the Reg-1 and Reg-2 regions are shown. Branch lengths represent the evolutionary time measured by the grids corresponding to the timescale shown at the tree base (in years). The tMRCA of selected nodes is reported with 95% confidence intervals. The time-frames of historical events mentioned in the text are reported

Data for both regions indicated that the earliest splits included the separation of enzootic and human-restricted genotypes (6795 to 4595 ya, depending on the region considered, plus confidence intervals), as well as the radiation of the camel-infecting genotypes 7 and 8 (6306–3431 ya) (Fig. 4). The most common genotypes were inferred to have appeared definitely earlier than previously estimated^7, 41, 42, 46^: the tMRCA of genotype 3 strains was estimated at 919–382 ya and genotype 4 originated 714–321 ya (Fig. 4). The analysis of HEV-3ra for Reg-1 is complicated by the presence of a highly divergent rabbit-derived sequence (accession: KJ013415) that, in analogy to previous analyses^47^, was basal to HEV-3 and HEV-3ra; excluding this sequence, the tMRCA of HEV-3 and HEV-3ra dates between 580 and 1436 CE (plus confidence intervals) (Fig. 4). The shallower tMRCA was observed for genotype 1, dating around 217–102 ya, whereas the split of the two human restricted genotypes occurred at least 4500 ya (Fig. 4).

### Dating the origin of the *Orthohepevirus* genus

Finally, we estimated the tMRCA of the *Orthohepevirus* genus using the same approach described above. To this aim, 31 *Orthohepevirus B* to *D* sequences, plus unclassified *Orthohepevirus*, were retrieved and included in the phylogeny with a subset of 82 *Orthohepevirus A* genomes (Supplementary Table S2). Due to poor alignment quality, a sub-region of Reg-1 (Reg-1-short, Fig. 1C) was used for dating.

Direct inference of the tMRCA with LSD was not possible due to extreme heterogeneity of branch lengths estimated by aBS-REL. We thus calculated branch lengths using a GTR model, and we used these lengths to estimate the tMRCA of extant Orthohepeviruses (Table 1). We next calculated the total branch length expansion of the aBS-REL model compared to the GTR model. This expansion was used to approximate the *Orthohepevirus* tMRCA. We observed a 1978 fold expansion of Reg-1-short and a 4621 fold expansion for Reg-2. The tMRCAs calculated using GTR branch lengths (Reg-1-short tMRCA: 24812 ya, Reg-2 tMRCA: 7300 ya) were thus adjusted by these expansions to obtain tMRCAs of ~49 million ya (IC: 21–56 million ya) for Reg-1-short and ~33 million ya (IC: 24–74 million ya) for Reg-2. As a consistency check, we used the same approach for *Orthohepevirus A* Reg-1 and Reg-2 and obtained comparable results to those obtained using LSD with aBS-REL branch lengths (Table 1).

## Discussion

HEV has a worldwide diffusion and causes a substantial health burden^48^. Investigating the origin and historical dissemination of this virus is important for understanding its present distribution and epidemic/pandemic potential.

Time inferences obtained using a selection-informed method were markedly different from those reported in other studies. Previous analyses focusing on the enzootic HEV strains dated the origin of genotype 4 in the Twentieth Century (1909, lower-bound estimate 1871)^46^, whereas different estimates were provided for genotype 3, with tMRCAs ranging from 320 to 199 ya^41, 42^. A study that analyzed HEV strains available in 2010 reached similar conclusions, with tMRCAs of 265–342 and 130–266 ya for genotypes 3 and 4, respectively^7^. In that analysis, the whole HEV phylogeny had a tMRCA of 536 ya, with a lower bound estimate of 1344 ya. The authors however noted that, because of sparse sampling and unaccounted variation in substitution rates, their dating strategy could have underestimated the true ages of these viral lineages^7^. Whereas the problem of incomplete sampling also applies to analyses herein, as several extant HEV lineages may remain undescribed and many others may have gone extinct, we applied a method that at least partially corrects for temporal variation in substitution rates. This approach was previously used to revise the evolutionary time frames of other RNA viruses, which resulted much deeper than those obtained with classical models^34, 45^. Although this method accounts for the effect of purifying selection, and we detected limited evidence of substitution saturation, we most likely failed to fully correct for time-dependent substitution rate variation^49^. As a consequence, the time frames we report may still underestimate the true timing of *Orthohepevirus* evolution.

Therefore, the methodology we applied for time inferences most likely accounts for the discrepancy between our findings and the previous ones^7, 41, 42, 46^. Indeed, when we performed molecular dating with models that do not account for selection (BEAST or LSD with GTR branch lengths), the tMRCA for the Orthohepevirus A phylogeny was not so different from that obtained by Purdy and coworkers^7^. Clearly, an additional source of diversity from that previous study derives from the dataset, as Purdy et al. performed their analyses in 2010, when several HEV genotypes (i.e., HEV-5 to HEV-8) had still to be discovered. As for phylogeography, our results are consistent with previous reports indicating a European origin for HEV-3^41, 42^. Conversely, data for HEV-4 are more difficult to interpret. Recently, this genotype was shown to have originated in Japan, although the skewed sampling of HEV-4 sequences was not accounted for^46^. We also obtained evidence of an East-Asian origin for this genotype using both BEAST and BBM. However, resampling with omission of character states for subsets of East-Asian strains yielded different results depending on the analyzed region and assigned HEV-4 to either West or South/South-East Asia. Overall, these data suggest that additional unbiased sampling of HEV-4 sequences will be required to correctly infer to geographic origin of this genotype (see also below).

Data herein indicate that *Orthohepevirus A* most likely originated in East Asia, from a human-infecting ancestor that existed ~6800 to ~4500 ya. This time frame corresponds to a period when intensive, sedentary agriculture (mainly rice and millet) was fully developed and spreading across China and neighboring regions^50–52^. The same period witnessed a rapid population growth in several East and South-East Asian regions and the appearance of relatively large human settlements^53^. These conditions, most probably characterized by the close proximity of habitation areas and waste deposits, as well as by the frequent contamination of drinking water^54^, may have facilitated the emergence of HEV strains with epidemiological features similar to extant human-restricted genotypes. Domesticated pigs were already present in China by 6000 BCE^55^, although husbandry practices probably intensified around 5000 to 3000 BCE^52, 56^. The close contact between humans and pigs was possibly responsible for the origin of the enzootic HEV strains, which represent one of the earliest splits in *Orthohepevirus A* phylogenies.

Our time estimates indicate that another early event in the *Orthohepevirus A* evolutionary history accompanied the emergence of camel-infecting genotypes. This observation is in line with a recent report that, based on HEV-7 genetic diversity and broad geographic distribution, suggested a long evolution of Orthohepevirus A in dromedary camels^57^. We dated the split of HEV-7 and HEV-8 from other genotypes in a wide time frame ranging from 4291 BCE to 1416 BCE (plus confidence intervals). This period encompasses the time of domestication of Bactrian camels (earliest evidences dating around 4500–3500 BCE) and dromedary camels (1000–2000 BCE)^58, 59^. The place and timing of HEV-3/HEV-3ra divergence also correspond remarkably well with the circumstances of rabbit domestication, which started in France around 600 CE^60^. However, the correspondence between domestication processes and divergence dates does not necessarily imply that the animal viruses originated from human transmission events. Husbandry practices commonly create animal colonies isolated from the other wildlife and living in crowded facilities. These conditions may have favored the spread of pre-existing animal viruses and their divergence into specific genotypes. With respect to phylogeographic analyses, we mention that an important limitation of our study is the underlying bias in sequence origin. We addressed this problem by both resampling with omission of character states for subsets of Asian strains and by analysis of an independent set of sequences which were selected to be equally representative of the continents. However, these approaches cannot compensate for areas where very few sequences were reported such as Central Asia and the Middle East. Sampling efforts in these regions will be pivotal to refine the geographic origin of HEV.

Finally, we note that the identification of humans as the likely ancestral hosts for *Orthohepevirus A* is consistent with the observation that most, if not all, HEV genotypes can infect our species. This is not merely a result of ecological factors, as inter-species transmission experiments confirmed the strict host specificity of HEV-1 and HEV-2^1^. Clearly, our inference concerning an ancestral human host for extant *Orthohepevirus A* strains does not exclude the possibility that humans acquired HEV through cross-species transmission from other animals. However, known Orthohepeviruses that infect mammals and birds are distantly related to *Orthohepevirus A*, suggesting that none of them represents the source of human-infecting HEV. To gain insight into the time of origin of the *Orthohepevirus* genus we exploited a strategy that was previously applied to Coronaviruses and is based on extrapolating over branch lengths obtained under different models^45^. In the case of Coronaviruses the authors obtained time estimates that were broadly consistent with the divergence time of birds and mammals, suggesting co-speciation of mammal-infecting and bird-infecting Coronaviruses with their hosts^45^. Conversely, the estimates we obtained for the tMRCA of the *Orthohepevirus* genus are much shallower than the divergence times of the host species that these viruses infect. Although these time frames may represent severe underestimates, the lack of virus-host co-speciation is also supported by the poor congruence of the viral and host phylogenies. Thus, ancient events of co-evolution and host-shifts probably contributed to originate extant *Orthohepevirus* species.

Several Orthohepeviruses have been described only recently^1^ and the current sampling of this viral genus is most likely limited in terms of host species. For instance, anti-HEV antibodies were detected in different animal species such as sheep, horses, cats, and dogs. Although no viral RNA has been isolated from these animals, it is likely that additional mammalian and non-mammalian HEV species or strains exist^1^. Analyses of more diverse potential hosts may thus shed further light into the ultimate origin of *Orthohepevirus A*.

## Electronic supplementary material

Supplementary Table S1

Supplementary Table S2

Supplementary Table S3

Supplementary Figure S1
