# Supplementary material for: Origin and dispersal of Hepatitis E virus
Source: Emerg Microbes Infect. 2018 Feb 7;7:11. doi: 10.1038/s41426-017-0009-6 (PMC5837148; doi:10.1038/s41426-017-0009-6)
Supplement: Supplementary file 1 — Supplementary Table S1 [file 41426_2017_9_MOESM1_ESM.doc]

**Supplementary Table S1** Geographic location of *Orthohepevirus A* genotypes

|  | **HEV-1** |  | **HEV-2** |  | **HEV-3** |  | **HEV-4** |  | **HEV-3ra** |  | **HEV-5** |  | **HEV-6** |  | **HEV-7** |  | **HEV-8** |
| --- | --- | --- | --- | --- | --- | --- | --- | --- | --- | --- | --- | --- | --- | --- | --- | --- | --- |
| **Asia** | Bangladesh, Cambodia, China, India, Israel, Japan, Kyrgyzstan, Iran, Myanmar, Nepal, Pakistan, Turkmenistan, Uzbekistan, Vietnam |  |  |  | Cambodia, China, India, Japan, Korea, Kyrgyzstan, Mongolia, Philippines, Singapore, Taiwan, Thailand |  | Cambodia, China, India, Indonesia, Japan, Korea, Mongolia, Taiwan, Vietnam |  | China |  | Japan |  | Japan |  | Pakistan, United Arab Emirates |  | China |
| **Africa** | Algeria, Central African Republic, Chad, Djibouti, Egypt, Ghana, Morocco, Namibia, South Africa, Sudan, Tunisia, Uganda |  | Central African Republic, Chad, Namibia, Democratic Republic of the Congo, Sudan, Tunisia |  | Burkina Faso, Cameroon, Central African Republic, Egypt, Madagascar, Mayotte, Nigeria, South Africa, Tunisia |  |  |  |  |  |  |  |  |  | Kenya, Somalia |  |  |
| **America** | Cuba, Venezuela |  | Mexico |  | Argentina, Bolivia, Brazil, Canada, Colombia, Costa Rica, Mexico, Uruguay, USA, Venezuela |  |  |  |  |  |  |  |  |  |  |  |  |
| **Europe** | Netherland, Russia, UK |  |  |  | Austria, Belgium, Czech Republic, Estonia, Finland, France, Germany, Greece, Hungary, Italy, Netherlands, Portugal, Romania, Russia, Spain, Sweden, Switzerland, UK |  | Denmark, France, Germany, Italy, Spain |  | France,  Netherlands |  |  |  |  |  |  |  |  |
| **Oceania** |  |  |  |  | Australia, New Zealand |  |  |  |  |  |  |  |  |  |  |  |  |

Note: HEV genotype geographic distributions were retrived from ^1-28^.

**References**

1. Aggarwal, R. Hepatitis e: epidemiology and natural history. *J Clin Exp Hepatol* 2013; **3:**125-133.

2. Okamoto, H. Genetic variability and evolution of hepatitis E virus. *Virus Res* 2007; **127:**216-228.

3. Geng, Y., Wang, Y. Epidemiology of Hepatitis E. *Adv Exp Med Biol* 2016; **948:**39-59.

4. Pauli, G., Aepfelbacher, M., Bauerfeind, U. *et al*. Hepatitis E Virus. *Transfus Med Hemother* 2015; **42:**247-265.

5. Echevarria, J. M., Gonzalez, J. E., Lewis-Ximenez, L. L. *et al*. Hepatitis E virus infection in Latin America: a review. *J Med Virol* 2013; **85:**1037-1045.

6. Kim, J. H., Nelson, K. E., Panzner, U., Kasture, Y., Labrique, A. B., Wierzba, T. F. Erratum to: A systematic review of the epidemiology of hepatitis E virus in Africa. *BMC Infect Dis* 2017; **17:**187-017-2274-3.

7. Kim, J. H., Nelson, K. E., Panzner, U., Kasture, Y., Labrique, A. B. & Wierzba, T. F. A systematic review of the epidemiology of hepatitis E virus in Africa. *BMC Infect Dis* 2014; **14:**308-2334-14-308.

8. Lorenzo, F. R., Tsatsralt-Od, B., Ganbat, S., Takahashi, M., Okamoto, H. Analysis of the full-length genome of hepatitis E virus isolates obtained from farm pigs in Mongolia. *J Med Virol* 2007; **79:**1128-1137.

9. Andersson, M. I., Preiser, W., Maponga, T. G. *et al*. Immune reconstitution hepatitis E: a neglected complication of antiretroviral therapy in Africa? *AIDS* 2013; **27:**487-489.

10. Pas, S. D., de Man, R. A., Mulders, C. *et al*. Hepatitis E virus infection among solid organ transplant recipients, the Netherlands. *Emerg Infect Dis* 2012; **18:**869-872.

11. Kokki, I., Smith, D., Simmonds, P. *et al*. Hepatitis E virus is the leading cause of acute viral hepatitis in Lothian, Scotland. *New Microbes New Infect* 2015; **10:**6-12.

12. Nakano, T., Takahashi, K., Takahashi, M. *et al*. Investigating the origin and global dispersal history of hepatitis E virus genotype 4 using phylogeographical analysis. *Liver Int* 2016; **36:**31-41.

13. Ivanova, A., Tefanova, V., Reshetnjak, I. *et al*. Hepatitis E Virus in Domestic Pigs, Wild Boars, Pig Farm Workers, and Hunters in Estonia. *Food Environ Virol* 2015; **7:**403-412.

14. Protzer, U., Bohm, F., Longerich, T. *et al*. Molecular detection of hepatitis E virus (HEV) in liver biopsies after liver transplantation. *Mod Pathol* 2015; **28:**523-532.

15. Mesquita, J. R., Oliveira, R. M., Coelho, C., Vieira-Pinto, M. & Nascimento, M. S. Hepatitis E Virus in Sylvatic and Captive Wild Boar from Portugal. *Transbound Emerg Dis*  2016; **63:**574-578.

16. Erez-Granat, O., Lachish, T., Daudi, N., Shouval, D. & Schwartz, E. Hepatitis E in Israel: A nation-wide retrospective study. *World J Gastroenterol* 2016; **22:**5568-5577.

17. Tsatsralt-Od, B., Baasanjav, N., Nyamkhuu, D., Ohnishi, H., Takahashi, M. & Okamoto, H. Prevalence of hepatitis viruses in patients with acute hepatitis and characterization of the detected genotype 4 hepatitis E virus sequences in Mongolia. *J Med Virol*  2016; **88:**282-291.

18. Liu, X., Saito, M., Sayama, Y. *et al*. Seroprevalence and molecular characteristics of hepatitis E virus in household-raised pig population in the Philippines. *BMC Vet Res*  2015; **11:**11-015-0322-z.

19. Yamada, H., Takahashi, K., Lim, O. *et al*. Hepatitis E Virus in Cambodia: Prevalence among the General Population and Complete Genome Sequence of Genotype 4. *PLoS One* 2015; **10:**e0136903.

20. Traore, K. A., Ouoba, J. B., Huot, N. *et al*. Hepatitis E Virus Exposure is Increased in Pork Butchers from Burkina Faso. *Am J Trop Med Hyg* 2015; **93:**1356-1359.

21. Forero, J. E., Gutierrez-Vergara, C., Parra Suescun, J. *et al*. Phylogenetic analysis of Hepatitis E virus strains isolated from slaughter-age pigs in Colombia. *Infect Genet Evol* 2017; **49:**138-145.

22. Parsa, R., Adibzadeh, S., Behzad Behbahani, A. *et al*. Detection of Hepatitis E Virus Genotype 1 Among Blood Donors From Southwest of Iran. *Hepat Mon* 2016; **16:**e34202.

23. Burt, S. A., Veltman, J., Hakze-van der Honing, R., Schmitt, H., van der Poel, W. H. Hepatitis E Virus in Farmed Rabbits, Wild Rabbits and Petting Farm Rabbits in the Netherlands. *Food Environ Virol* 2016; **8:**227-229.

24. Doceul, V., Bagdassarian, E., Demange, A. & Pavio, N. Zoonotic Hepatitis E Virus: Classification, Animal Reservoirs and Transmission Routes. *Viruses* 2016; **8:**E270.

25. Rasche, A., Saqib, M., Liljander, A. M. *et al*. Hepatitis E Virus Infection in Dromedaries, North and East Africa, United Arab Emirates, and Pakistan, 1983-2015. *Emerg Infect Dis* 2016; **22:**1249-1252.

26. Woo, P. C., Lau, S. K., Teng, J. L. *et al*. New Hepatitis E Virus Genotype in Bactrian Camels, Xinjiang, China, 2013. *Emerg Infect Dis* 2016; **22:**2219-2221.

27. Takahashi, M., Nishizawa, T., Sato, H. *et al*. Analysis of the full-length genome of a hepatitis E virus isolate obtained from a wild boar in Japan that is classifiable into a novel genotype. *J Gen Virol* 2011; **92:**902-908.

28. Takahashi, M., Nishizawa, T., Nagashima, S. *et al*. Molecular characterization of a novel hepatitis E virus (HEV) strain obtained from a wild boar in Japan that is highly divergent from the previously recognized HEV strains. *Virus Res* 2014; **180:**59-69.
