# Supplementary material for: Origin and dispersal of Hepatitis E virus
Source: Emerg Microbes Infect. 2018 Feb 7;7:11. doi: 10.1038/s41426-017-0009-6 (PMC5837148; doi:10.1038/s41426-017-0009-6)
Supplement: Supplementary file 2 — Supplementary Table S2 [file 41426_2017_9_MOESM2_ESM.doc]

**Supplementary Table S2**. List of analyzed sequences

| **Strain Name** | **Species** | **Genbank ID** | **Collection Date** | **Host** | **Country** | **Region** | ***Orthohepevirus A* analyses** | ***Orthohepevirus* genus dating** |
| --- | --- | --- | --- | --- | --- | --- | --- | --- |
| ***Orthohepevirus*** |  |  |  |  |  |  |  |  |
| swJ570 | A | AB073912 | 2001 | Swine | Japan | EA | * |  |
| JAK-Sai | A | AB074915 | 2001 | Human | Japan | EA | * | * |
| HE-JI4 | A | AB080575 | 2000 | Human | Japan | EA | * |  |
| HE-JA10 | A | AB089824 | 1993 | Human | Japan | EA | * | * |
| swJ13-1 | A | AB097811 | 2002 | Swine | Japan | EA | * |  |
| HE-JA1 | A | AB097812 | 1997 | Human | Japan | EA | * |  |
| HE-JK4 | A | AB099347 | 2002 | Human | Japan | EA | * |  |
| CCC220 | A | AB108537 | 2000 | Human | China | EA | * | * |
| JSM-Sap95 | A | AB161717 | 1995 | Human | Japan | EA | * | * |
| JTS-Sap02 | A | AB161718 | 2002 | Human | Japan | EA | * |  |
| JYW-Sap02 | A | AB161719 | 2002 | Human | Japan | EA | * |  |
| JBOAR1-Hyo04 | A | AB189070 | 2004 | Boar | Japan | EA | * |  |
| JDEER-Hyo03L | A | AB189071 | 2003 | Deer | Japan | EA | * |  |
| JMO-Hyo03L | A | AB189072 | 2003 | Human | Japan | EA | * |  |
| JSO-Hyo03L | A | AB189073 | 2003 | Human | Japan | EA | * |  |
| JTH-Hyo03L | A | AB189074 | 2003 | Human | Japan | EA | * |  |
| JYO-Hyo03L | A | AB189075 | 2003 | Human | Japan | EA | * |  |
| JSF-Tot03C | A | AB193176 | 2003 | Human | Japan | EA | * | * |
| JYN-Sap01C | A | AB193177 | 2001 | Human | Japan | EA | * |  |
| JYN-Nii02L | A | AB193178 | 2002 | Human | Japan | EA | * |  |
| JKO-ChiSai98C | A | AB197673 | 1998 | Human | China | EA | * | * |
| JSN-Sap-FH02C | A | AB200239 | 2002 | Human | Japan | EA | * |  |
| HE-JA2 | A | AB220974 | 1998 | Human | Japan | EA | * | * |
| wbJSG1 | A | AB222182 | 2004 | Boar | Japan | EA | * |  |
| wbJTS1 | A | AB222183 | 2004 | Boar | Japan | EA | * |  |
| wbJYG1 | A | AB222184 | 2004 | Boar | Japan | EA | * |  |
| JMNG-Oki02C | A | AB236320 | 2002 | Mongoose | Japan | EA | * | * |
| HE-JA04-1911 | A | AB248520 | 2004 | Human | Japan | EA | * |  |
| swJ8-5 | A | AB248521 | 2004 | Swine | Japan | EA | * | * |
| swJ12-4 | A | AB248522 | 2004 | Swine | Japan | EA | * |  |
| AB290312 | A | AB290312 | 2006 | Swine | Mongolia | EA | * | * |
| AB290313 | A | AB290313 | 2006 | Swine | Mongolia | EA | * | * |
| JIO-Sai97L | A | AB291951 | 1997 | Human | Japan | EA | * |  |
| JIY-Tot05L | A | AB291952 | 2005 | Human | Japan | EA | * |  |
| JSO-Oki05L | A | AB291953 | 2005 | Human | Japan | EA | * |  |
| JSS-Oka04L | A | AB291954 | 2004 | Human | Japan | EA | * |  |
| JSW-Kyo-FH06L | A | AB291955 | 2006 | Human | Japan | EA | * |  |
| JYM-Tot04L | A | AB291956 | 2004 | Human | Japan | EA | * |  |
| JYU-Oki04L | A | AB291957 | 2004 | Human | Japan | EA | * |  |
| JNH-Ehi04L | A | AB291958 | 2004 | Human | Japan | EA | * |  |
| JTC-Kit-FH04L | A | AB291959 | 2004 | Human | Japan | EA | * |  |
| JTK-Kag06C | A | AB291960 | 2006 | Human | Japan | EA | * |  |
| JMH-Osa04C | A | AB291961 | 2004 | Human | Japan | EA | * |  |
| JHK-Toy04C | A | AB291962 | 2004 | Human | Japan | EA | * |  |
| JRM-Toy05C | A | AB291963 | 2005 | Human | Japan | EA | * |  |
| JYK-Tok03C | A | AB291964 | 2003 | Human | Japan | EA | * |  |
| HRC-HE14C | A | AB291965 | 2004 | Human | Japan | EA | * |  |
| JST-KitAsa04C | A | AB291966 | 2004 | Human | Japan | EA | * |  |
| JKO-Aba-FH06C | A | AB291967 | 2006 | Human | Japan | EA | * |  |
| JMM-Aba06C | A | AB291968 | 2006 | Human | Japan | EA | * |  |
| JE03-1760F | A | AB301710 | 2003 | Human | Japan | EA | * |  |
| E116-YKH98C | A | AB369687 | 1998 | Human | Thailand | S/SE Asia | * | * |
| E087-SAP04C | A | AB369688 | 2004 | Human | Japan | EA | * | * |
| E088-STM04C | A | AB369689 | 2004 | Human | Japan | EA | * | * |
| E067-SIJ05C | A | AB369690 | 2005 | Human | Japan | EA | * |  |
| E097-OSA05C | A | AB369691 | 2005 | Human | Japan | EA | * |  |
| JIO-swJ19-1 | A | AB443623 | 2002 | Swine | Japan | EA | * |  |
| IO-swJ19-2 | A | AB443624 | 2002 | Human | Japan | EA | * |  |
| IO-swJ19-5 | A | AB443625 | 2002 | Swine | Japan | EA | * |  |
| IO-swJ19-7 | A | AB443626 | 2002 | Swine | Japan | EA | * |  |
| IO-swJ19-8 | A | AB443627 | 2002 | Swine | Japan | EA | * |  |
| swJR-P5 | A | AB481229 | 2009 | Swine | Japan | EA | * |  |
| JKS-Shiz07L | A | AB521805 | 2007 | Human | Japan | EA | * |  |
| JYN-Shiz08L | A | AB521806 | 2008 | Human | Japan | EA | * |  |
| JBOAR135-Shiz09 | A | AB573435 | 2009 | Swine | Japan | EA | * | * |
| JMNG26-Oki08 | A | AB591733 | 2008 | Mongoose | Japan | EA | * |  |
| JMNG36-Oki08 | A | AB591734 | 2008 | Mongoose | Japan | EA | * |  |
| HE-Aichi-C1 | A | AB602439 | 2005 | Human | Japan | EA | * |  |
| wbJGF_08-1 | A | AB602440 | 2008 | Swine | Japan | EA | * |  |
| wbJOY_06 | A | AB602441 | 2006 | Swine | Japan | EA | * | * |
| HRC-HE104 | A | AB630970 | 2007 | Human | Japan | EA | * |  |
| JRC-HE3 | A | AB630971 | 2003 | Human | Japan | EA | * | * |
| Type III Highland/2000 | A | AB698071 | 2000 | Swine | Japan | EA | * |  |
| JTF-Yamagu11 | A | AB698654 | 2011 | Human | Japan | EA | * |  |
| E11-Ban10 | A | AB720034 | 2010 | Human | Bangladesh | S/SE Asia | * | * |
| E13-Ban10 | A | AB720035 | 2010 | Human | Bangladesh | S/SE Asia | * |  |
| AB740220 | A | AB740220 | 2011 | Rabbit | China | EA | * | * |
| AB740221 | A | AB740221 | 2011 | Rabbit | China | EA | * |  |
| AB740222 | A | AB740222 | 2011 | Rabbit | China | EA | * |  |
| JBOAR100-Mie10 | A | AB780450 | 2010 | Swine | Japan | EA | * |  |
| JBOAR107-Mie11 | A | AB780451 | 2011 | Swine | Japan | EA | * |  |
| JBOAR111-Mie11 | A | AB780452 | 2011 | Swine | Japan | EA | * |  |
| JBOAR124-Mie11 | A | AB780453 | 2011 | Swine | Japan | EA | * | * |
| HE-JA12-0725 | A | AB850879 | 2012 | Human | Japan | EA | * | * |
| wbJNN_13 | A | AB856243 | 2013 | Swine | Japan | EA | * | * |
| JAO-Gif12 | A | AB909124 | 2012 | Human | Japan | EA | * |  |
| JKK-Shiz13 | A | AB909125 | 2013 | Human | Japan | EA | * |  |
| TK15/92 | A | AF051830 | 1998 | Human | Nepal | S/SE Asia | * | * |
| HEV-US2 | A | AF060669 | 1998 | Human | USA | NoA | * | * |
| AF076239 | A | AF076239 | 1998 | Human | India | S/SE Asia | * | * |
| Meng | A | AF082843 | 1997 | Swine | USA | NoA | * | * |
| Osh 205 | A | AF455784 | 2003 | Swine | Kyrgyzstan | EA | * | * |
| AF459438 | A | AF459438 | 1989 | Human | India | S/SE Asia | * |  |
| T1 | A | AJ272108 | 1997 | Human | China | EA | * | * |
| JRA1 | A | AP003430 | 2001 | Human | Japan | EA | * | * |
| Arkell | A | AY115488 | 2001 | Swine | Canada | NoA | * | * |
| T3 | A | AY204877 | 1983 | Human | Chad | AF | * | * |
| Morocco_1D | A | AY230202 | 2003 | Human | Morocco | AF | * | * |
| IND-SW-00-01 | A | AY723745 | 2000 | Swine | India | S/SE Asia | * | * |
| HEVNE8L | A | D10330 | 1992 | Human | Myanmar | S/SE Asia | * |  |
| HPECG_1b | A | D11092 | 1987 | Human | China | EA | * | * |
| swDQ | A | DQ279091 | 2007 | Swine | China | EA | * | * |
| swCH31 | A | DQ450072 | 2006 | Swine | China | EA | * | * |
| UNKNOWN-DQ459342 | A | DQ459342 | 2000 | Human | India | S/SE Asia | * |  |
| swX07-E1 | A | EU360977 | 2007 | Swine | Sweden | EU | * | * |
| Thai-swHEV07 | A | EU375463 | 2006 | Swine | Thailand | S/SE Asia | * | * |
| SW626 | A | EU723512 | 2008 | Swine | Spain | EU | * |  |
| SW627 | A | EU723513 | 2008 | Swine | Spain | EU | * | * |
| SWP6 | A | EU723514 | 2009 | Swine | Spain | EU | * | * |
| SWP7 | A | EU723515 | 2008 | Swine | Spain | EU | * |  |
| SWP8 | A | EU723516 | 2008 | Swine | Spain | EU | * |  |
| swKOR-1 | A | FJ426403 | 2007 | Swine | South Korea | EA | * |  |
| swKOR-2 | A | FJ426404 | 2007 | Swine | South Korea | EA | * |  |
| HEV-H | A | FJ457024 | 2005 | Human | India | S/SE Asia | * |  |
| SAAS-JDY5 | A | FJ527832 | 2008 | Swine | China | EA | * |  |
| swCH189 | A | FJ610232 | 2008 | Swine | China | EA | * | * |
| CU001 | A | FJ653660 | 2008 | Human | Thailand | S/SE Asia | * |  |
| wbGER27 | A | FJ705359 | 2006 | Boar | Germany | EU | * | * |
| KNIH-hHEV4 | A | FJ763142 | 2007 | Human | South Korea | EA | * | * |
| GDC9 | A | FJ906895 | 2009 | Rabbit | China | EA | * | * |
| GDC46 | A | FJ906896 | 2009 | Rabbit | China | EA | * |  |
| HEV_RKI | A | FJ956757 | 2005 | Human | Germany | EU | * | * |
| BB02 | A | FJ998008 | 2007 | Swine | Germany | EU | * | * |
| CHN-XJ-SW33 | A | GU119960 | 2009 | Swine | China | EA | * |  |
| CHN-XJ-SW13 | A | GU119961 | 2009 | Swine | China | EA | * | * |
| WH09 | A | GU188851 | 2009 | Swine | China | EA | * |  |
| bjsw1 | A | GU206559 | 2008 | Swine | China | EA | * |  |
| hb-3 | A | GU361892 | 2008 | Swine | China | EA | * |  |
| ch-bj-n1 | A | GU937805 | 2009 | Rabbit | China | EA | * |  |
| EChZ20 | A | HM439284 | 2008 | Human | China | EA | * |  |
| IND-HEV-AVH1-1991_1F | A | JF443717 | 1991 | Human | India | S/SE Asia | * |  |
| IND-HEV-AVH2-1998 | A | JF443718 | 1998 | Human | India | S/SE Asia | * | * |
| IND-HEV-AVH3-2000 | A | JF443719 | 2000 | Human | India | S/SE Asia | * |  |
| IND-HEV-AVH4-2006 | A | JF443720 | 2006 | Human | India | S/SE Asia | * | * |
| IND-HEV-AVH5-2010 | A | JF443721 | 2010 | Human | India | S/SE Asia | * | * |
| IND-HEV-FHF1-2003 | A | JF443722 | 2003 | Human | India | S/SE Asia | * |  |
| IND-HEV-FHF2-2004 | A | JF443723 | 2004 | Human | India | S/SE Asia | * | * |
| IND-HEV-FHF3-2005 | A | JF443724 | 2005 | Human | India | S/SE Asia | * | * |
| IND-HEV-FHF4-2006 | A | JF443725 | 2006 | Human | India | S/SE Asia | * |  |
| IND-HEV-FHF5-2007 | A | JF443726 | 2007 | Human | India | S/SE Asia | * | * |
| SAAS-FX17 | A | JF915746 | 2009 | Swine | China | EA | * | * |
| FR-HuHEVF3f | A | JN906974 | 2010 | Human | France | EU | * |  |
| FR-SHEVF3f | A | JN906975 | 2010 | Swine | France | EU | * |  |
| FR-SHEVB3f | A | JN906976 | 2010 | Swine | France | EU | * |  |
| W1-11 | A | JQ013791 | 2007 | Rabbit | France | EU | * | * |
| W7-57 | A | JQ013792 | 2007 | Rabbit | France | EU | * |  |
| TLS-18516 | A | JQ013793 | 2008 | Human | France | EU | * | * |
| TR19 | A | JQ013794 | 2007 | Human | France | EU | * | * |
| TR02 | A | JQ013795 | 2006 | Human | France | EU | * |  |
| Inuyama | A | JQ026407 | 2009 | Crab-eating Macaque | Japan | EA | * |  |
| MO | A | JQ655733 | 2006 | Human | China | EA | * |  |
| W3 | A | JQ655735 | 2006 | Human | China | EA | * |  |
| W2-5 | A | JQ655736 | 2006 | Swine | China | EA | * |  |
| CHN-NJ-H2011 | A | JQ740781 | 2009 | Human | China | EA | * |  |
| FR-SHEV3c-like | A | JQ953664 | 2006 | Swine | France | EU | * | * |
| FR-SHEV3e | A | JQ953665 | 2006 | Swine | France | EU | * |  |
| FR-SHEV3f | A | JQ953666 | 2008 | Swine | France | EU | * |  |
| HEV-ZJ1 | A | JQ993308 | 2009 | Swine | China | EA | * |  |
| CMC-1 | A | JX565469 | 2010 | Rabbit | USA | NoA | * | * |
| SS19 | A | JX855794 | 2011 | Swine | China | EA | * |  |
| CH-YT-1 | A | KC163335 | 2012 | Human | China | EA | * |  |
| CH-YT-HEV02 | A | KC492825 | 2011 | Human | China | EA | * | * |
| CH-YT-sHEV01 | A | KC692453 | 2011 | Swine | China | EA | * |  |
| CHN-SD-sHEV | A | KF176351 | 2011 | Swine | China | EA | * |  |
| CHN-QH-Yak27 | A | KF736234 | 2013 | Yak | China | EA | * | * |
| AlgSwe2012 |  | KF951328 | 2012 | Moose | Sweden | EU | * | * |
| CHN-BJ-r14(9) | A | KJ013415 | 2013 | Rabbit | China | EA | * | * |
| KM01 | A | KJ155502 | 2010 | Swine | China | EA | * | * |
| 178C | A | KJ496143 | 2013 | Camel | United Arab Emirates | WeA | * | * |
| 180C | A | KJ496144 | 2013 | Camel | United Arab Emirates | WeA | * | * |
| swSTHY12-VAS19/2003/CA | A | KJ507955 | 2003 | Swine | Canada | NoA | * |  |
| swSTHY42-VAS49/2003/CA | A | KJ507956 | 2003 | Swine | Canada | NoA | * |  |
| MWP_2010 | A | KP294371 | 2010 | Boar | Germany | EU | * |  |
| SwHEVE2IT12 | A | KP698919 | 2012 | Swine | Italy | EU | * |  |
| KM03 | A | KR872414 | 2014 | Rhesus Macaque | China | EA | * | * |
| KM05 | A | KR872415 | 2015 | Human | China | EA | * |  |
| KM02 | A | KR872416 | 2014 | Shrew | China | EA | * | * |
| KM04 | A | KR872417 | 2015 | Human | China | EA | * |  |
| Sing-HEV01 | A | KT447526 | 2010 | Human | Singapore | S/SE Asia | * | * |
| Sing-HEV20 | A | KT447527 | 2011 | Human | Singapore | S/SE Asia | * |  |
| Sing-HEV23 | A | KT447528 | 2012 | Human | Singapore | S/SE Asia | * |  |
| ZhJ-PJ050-3 | A | KT633715 | 2011 | Swine | China | EA | * |  |
| CCST-517 | A | KT727028 | 2015 | Swine | China | EA | * | * |
| Goat-HEV-5 | A | KU356182 | 2015 | Goat | China | EA | * |  |
| Goat-HEV-9 | A | KU356183 | 2015 | Goat | China | EA | * |  |
| Goat-HEV-18 | A | KU356184 | 2015 | Goat | China | EA | * | * |
| Goat-HEV-42 | A | KU356185 | 2015 | Goat | China | EA | * |  |
| Goat-HEV-52 | A | KU356186 | 2015 | Goat | China | EA | * |  |
| cow-34 | A | KU356187 | 2015 | Cattle | China | EA | * | * |
| cow-3 | A | KU356188 | 2015 | Cattle | China | EA | * |  |
| cow-24 | A | KU356189 | 2015 | Cattle | China | EA | * |  |
| CHN-SX-rHEV | A | KX227751 | 2015 | Rabbit | China | EA | * | * |
| 12XJ | A | KX387865 | 2013 | Camel | China | EA | * |  |
| 48XJ | A | KX387866 | 2013 | Camel | China | EA | * | * |
| 62XJ | A | KX387867 | 2013 | Camel | China | EA | * | * |
| K52-87 | A | L25595 | 1987 | Human | China | EA | * |  |
| HE-JA30 | A | LC022745 | 2003 | Human | Japan | EA | * |  |
| MNE13-227 | A | LC037955 | 2013 | Human | Mongolia | EA | * |  |
| CVS-Sie10 | A | LC042232 | 2012 | Human | Cambodia | S/SE Asia | * | * |
| JAO-SpaTok12 | A | LC055972 | 2012 | Human | Japan | EA | * |  |
| JMH-Sai14 | A | LC055973 | 2014 | Human | Japan | EA | * |  |
| Burma_1a | A | M73218 | 1991 | Human | Myanmar | S/SE Asia | * | * |
| m1_2A | A | M74506 | 1992 | Human | Mexico | CeA | * | * |
| Sar-55 | A | M80581 | 1987 | Human | Pakistan | S/SE Asia | * | * |
| HPEGENSA | A | NC_001434 | 1987 | Human | China | EA | * | * |
| I1_1c | A | X98292 | 1996 | Human | India | S/SE Asia | * | * |
| individual patient infected with HEV | A | X99441 | 1993 | Human | India | S/SE Asia | * | * |
| 05-5492 | B | AM943646 | 2005 | Chicken | Australia | Oceania |  | * |
| 06-561 | B | AM943647 | 1986 | Chicken | Australia | Oceania |  | * |
| EF206691 | B | EF206691 | 2004 | Chicken | USA | NoA |  | * |
| CaHEV | B | GU954430 | 2009 | Chicken | China | EA |  | * |
| HH-F9 | B | JN597006 | 2011 | Chicken | South Korea | EA |  | * |
| HU-16773 | B | JN997392 | 2010 | Chicken | Hungary | EU |  | * |
| JY-F2 | B | KC454286 | 2011 | Chicken | South Korea | EA |  | * |
| KF511797 | B | KF511797 | 2012 | Chicken | Taiwan | S/SE Asia |  | * |
| GI-B | B | KM377618 | 2014 | Chicken | South Korea | EA |  | * |
| NC_023425 | B | NC_023425 | 2001 | Chicken | USA | NoA |  | * |
| ratIDE079F | C | AB847305 | 2011 | Rat | Indonesia | S/SE Asia |  | * |
| ratESOLO-014SF | C | AB847306 | 2012 | Rat | Indonesia | S/SE Asia |  | * |
| ratELOMB-187SF | C | AB847307 | 2012 | Rat | Indonesia | S/SE Asia |  | * |
| ratESOLO-006SF | C | AB847308 | 2012 | Rat | Indonesia | S/SE Asia |  | * |
| ratIDE113F | C | AB847309 | 2012 | Rat | Indonesia | S/SE Asia |  | * |
| HEV-4351 | C | AB890001 | 2013 | Ferret | Japan | EA |  | * |
| HEV-4342 | C | AB890374 | 2013 | Ferret | Japan | EA |  | * |
| R63 | C | GU345042 | 2009 | Rat | Germany | EU |  | * |
| R68 | C | GU345043 | 2009 | Rat | Germany | EA |  | * |
| rat/Mu09/0685/DEU/2010 | C | JN167537 | 2009 | Rat | Germany | EU |  | * |
| rat/Mu09/0434/DEU/2010 | C | JN167538 | 2009 | Rat | Germany | EU |  | * |
| FRHEV4 | C | JN998606 | 2010 | Polecat | Netherlands | EU |  | * |
| FRHEV20 | C | JN998607 | 2010 | Polecat | Netherlands | EU |  | * |
| Vietnam-105 | C | JX120573 | 2011 | Rat | Viet Nam | S/SE Asia |  | * |
| LA-B350 | C | KM516906 | 2003 | Rat | USA | NoA |  | * |
| ratELOMB-131 | C | LC145325 | 2012 | Rat | Indonesia | S/SE Asia |  | * |
| F45 | C | LC177788 | 2016 | Ferret | USA | NoA |  | * |
| F54 | C | LC177789 | 2016 | Ferret | USA | NoA |  | * |
| F60 | C | LC177790 | 2016 | Ferret | USA | NoA |  | * |
| Rf-HEV/Shanxi2013 | D | KJ562187 | 2013 | Bat | China | EA |  | * |
| BatHEV/BS7/GE/2009 | D | NC_018382 | 2009 | Bat | Germany | EU |  | * |
| kestrel/MR22/2014/HUN |  | KU670940 | 2014 | Common Kestrel | Hungary | EU |  | * |
| little egret/kocsag02/2014/HUN |  | KX589065 | 2014 | Little egret | Hungary | EU |  | * |
|  |  |  |  |  |  |  |  |  |
| ***Piscihepevirus*** |  |  |  |  |  |  |  |  |
| Heenan88 |  | NC_015521 | 1988 | Cutthroat Trout | USA | NoA |  | * |

AF: Africa, EA: East Asia, EU: Europe, CeA: Central America, NoA: North America, WeA: West Asia, S/SE Asia: South/South-East Asia
