# Supplementary material for: Origin and dispersal of Hepatitis E virus
Source: Emerg Microbes Infect. 2018 Feb 7;7:11. doi: 10.1038/s41426-017-0009-6 (PMC5837148; doi:10.1038/s41426-017-0009-6)
Supplement: Supplementary file 3 — Supplementary Table S3 [file 41426_2017_9_MOESM3_ESM.doc]

**Supplementary Table S3**. List of sequences used for the Reg-2-short phylogeographic analysis

| **Strain Name** | **GenBank ID** | **Collection Date** | **Host** | **Country** | **Region** |
| --- | --- | --- | --- | --- | --- |
| 08/126 | JN863910 | 2008 | Human | Central African Republic | Africa |
| 08/170 | JN863909 | 2008 | Human | Central African Republic | Africa |
| CAR_08/419 | KF734744 | 2008 | Human | Central African Republic | Africa |
| CAR_08/336 | KF734739 | 2008 | Human | Central African Republic | Africa |
| CAR_08/338 | KF734740 | 2008 | Human | Central African Republic | Africa |
| CAR_08/346 | KF734742 | 2008 | Human | Central African Republic | Africa |
| CAR_08/418 | KF734743 | 2008 | Human | Central African Republic | Africa |
| CAR_08/344 | KF734741 | 2008 | Human | Central African Republic | Africa |
| CAR_08/432 | KF734745 | 2008 | Human | Central African Republic | Africa |
| CAR_09/695 | KF734753 | 2009 | Human | Central African Republic | Africa |
| CAR_09/251 | KF734748 | 2009 | Human | Central African Republic | Africa |
| CAR_09/212 | KF734760 | 2009 | Human | Central African Republic | Africa |
| CAR_09/248 | KF734761 | 2009 | Human | Central African Republic | Africa |
| CAR_09/249 | KF734751 | 2009 | Human | Central African Republic | Africa |
| CAR_09/671 | KF734750 | 2009 | Human | Central African Republic | Africa |
| CAR_09/226 | KF734746 | 2009 | Human | Central African Republic | Africa |
| CAR_09/240 | KF734747 | 2009 | Human | Central African Republic | Africa |
| CAR_09/733 | KF734755 | 2009 | Human | Central African Republic | Africa |
| CAR_09/764 | KF734759 | 2009 | Human | Central African Republic | Africa |
| CAR_09/685 | KF734752 | 2009 | Human | Central African Republic | Africa |
| CAR_09/700 | KF734754 | 2009 | Human | Central African Republic | Africa |
| CAR_09/317 | KF734749 | 2009 | Human | Central African Republic | Africa |
| CAR_09/754 | KF734757 | 2009 | Human | Central African Republic | Africa |
| CAR_09/735 | KF734756 | 2009 | Human | Central African Republic | Africa |
| CAR_09/758 | KF734758 | 2009 | Human | Central African Republic | Africa |
| CAR_09/744 | KF734762 | 2009 | Human | Central African Republic | Africa |
| 11/289 | JQ740782 | 2011 | Human | Central African Republic | Africa |
| 11/009 | JQ074213 | 2011 | Human | Central African Republic | Africa |
| 11/176 | JN863908 | 2011 | Human | Central African Republic | Africa |
| CPBA0407028 | KP404612 | 2013 | Human | Central African Republic | Africa |
| T3 | AY204877 | 1983 | Human | Chad | Africa |
| 93-Egypt | AF051351 | 1993 | Human | Egypt | Africa |
| 94-Egypt | AF051352 | 1993 | Human | Egypt | Africa |
| LAHIV_G3 | KU178915 | 2014 | Human | South Africa | Africa |
| WBRENAL_G3 | KU178916 | 2014 | Human | South Africa | Africa |
| SCH64333X | KT833800 | 2013 | Human | South Africa | Africa |
| LN831924 | LN831924 | 2013 | Swine | Burkina Faso | Africa |
| Yaounde56 | KC012634 | 2012 | Swine | Cameroon | Africa |
| Yaounde94 | KC012635 | 2012 | Swine | Cameroon | Africa |
| Mars-Kins17 | FJ600536 | 2008 | Swine | Democratic Republic of the Congo | Africa |
| swHEV-MG-104 | JX507128 | 2010 | Swine | Madagascar | Africa |
| swHEV-MG-121 | JX507129 | 2010 | Swine | Madagascar | Africa |
| swHEV-MG-190 | JX507130 | 2010 | Swine | Madagascar | Africa |
| swHEV_LS13_Nig11 | KJ451630 | 2011 | Swine | Nigeria | Africa |
| swHEV_MG18_Nig11 | KJ451629 | 2011 | Swine | Nigeria | Africa |
| swHEV_MG25_Nig11 | KJ451632 | 2011 | Swine | Nigeria | Africa |
| swHEV_SHD50_Nig11 | KJ451631 | 2011 | Swine | Nigeria | Africa |
| swHEV_MG7_Nig11 | KJ451633 | 2011 | Swine | Nigeria | Africa |
| Milk15/1/Kunming | KU974933 | 2016 | Cattle | China | Asia |
| HuYN-01 | HQ828107 | 2009 | Human | China | Asia |
| JZH10 | KF691601 | 2012 | Human | China | Asia |
| JE03-1760F | AB437316 | 2003 | Human | Japan | Asia |
| HE-JA07-0744 | AB971729 | 2007 | Human | Japan | Asia |
| HE-JA13-2014 | AB971744 | 2013 | Human | Japan | Asia |
| HE-JA14-0488 | LC022744 | 2014 | Human | Japan | Asia |
| MNE13-242_ORF2 | LC037969 | 2013 | Human | Mongolia | Asia |
| E01-Ban10 | AB720036 | 2010 | Human | Bangladesh | Asia |
| E02-Ban10 | AB720037 | 2010 | Human | Bangladesh | Asia |
| E06-Ban10 | AB720041 | 2010 | Human | Bangladesh | Asia |
| E08-Ban10 | AB720043 | 2010 | Human | Bangladesh | Asia |
| E04-Ban10 | AB720039 | 2010 | Human | Bangladesh | Asia |
| E09-Ban10 | AB720044 | 2010 | Human | Bangladesh | Asia |
| E14-Ban10 | AB720047 | 2010 | Human | Bangladesh | Asia |
| E15-Ban10 | AB720048 | 2010 | Human | Bangladesh | Asia |
| 20 | KR027517 | 2004 | Human | India | Asia |
| 222 | KR027542 | 2012 | Human | India | Asia |
| 259 | KR027582 | 2012 | Human | India | Asia |
| 1 | KR185381 | 2013 | Human | India | Asia |
| 331 | KR027662 | 2013 | Human | India | Asia |
| 642 | KR027161 | 2014 | Human | India | Asia |
| 561 | KR027818 | 2014 | Human | India | Asia |
| 544 | KR027799 | 2014 | Human | India | Asia |
| Mya86-Li | DQ079624 | 2005 | Human | Myanmar | Asia |
| Patient 17 | KT818608 | 2011 | Human | Singapore | Asia |
| HEV2-CU22 | JX841316 | 2011 | Human | Thailand | Asia |
| rbIM025-c1 | AB741096 | 2009 | Rabbit | China | Asia |
| CHN-BJ-RC2 | KP663376 | 2015 | Rabbit | China | Asia |
| HB-RD1 | JX501274 | 2012 | Rabbit | China | Asia |
| CHN-BJ-R14 | JX109834 | 2011 | Rabbit | China | Asia |
| CHN-BJ-SPF10 | KU217471 | 2015 | Rabbit | China | Asia |
| CHN-TJ-B3 | KJ648609 | 2014 | Swine | China | Asia |
| HB-SB | JX501258 | 2012 | Swine | China | Asia |
| CHN-LYG-SJ13 | KF564272 | 2013 | Swine | China | Asia |
| KMsw-1 | HQ008863 | 2010 | Swine | China | Asia |
| CHN-HN-SW22 | KP284147 | 2014 | Swine | China | Asia |
| HB-SG2 | JX501266 | 2012 | Swine | China | Asia |
| HB-SD2 | JX501261 | 2012 | Swine | China | Asia |
| CHN-LYG-SJ3 | KF564262 | 2013 | Swine | China | Asia |
| CHN-HB-HD-L5 | KJ534656 | 2013 | Swine | China | Asia |
| genotype 3 | KF719310 | 2013 | Swine | Japan | Asia |
| JSW.H2-Oki08 | AB919134 | 2008 | Swine | Japan | Asia |
| JBOAR012-Mie08 | AB780455 | 2008 | Swine | Japan | Asia |
| HEV/Japan-Okazaki/1412boar077 | LC075751 | 2014 | Swine | Japan | Asia |
| HEV/Japan-Okazaki/1301boar073 | LC075750 | 2013 | Swine | Japan | Asia |
| JBOAR120-Mie11 | AB780457 | 2011 | Swine | Japan | Asia |
| JWB.C76-Iri10 | AB919132 | 2010 | Swine | Japan | Asia |
| BeWbL1 | KP296181 | 2010 | Boar | Belgium | Europe |
| 282 MRS | FJ718694 | 2013 | Boar | France | Europe |
| wbGER27 | FJ705359 | 2006 | Boar | Germany | Europe |
| MWP_2010 | KP294371 | 2010 | Boar | Germany | Europe |
| WB55 | KJ567079 | 2012 | Boar | Italy | Europe |
| BeCL48 | KR149812 | 2012 | Deer | Belgium | Europe |
| HEV/Goat/PB29/ITA | KX470598 | 2016 | Goat | Italy | Europe |
| BeHu1 | KC961251 | 2009 | Human | Belgium | Europe |
| BeHu10 | KC961259 | 2012 | Human | Belgium | Europe |
| Dk_Donor4 | KU702729 | 2015 | Human | Denmark | Europe |
| 12F29985 | KC928081 | 2012 | Human | Denmark | Europe |
| 8 | KR027405 | 2003 | Human | France | Europe |
| Fr-09AK47 | JF730423 | 2009 | Human | France | Europe |
| HEV-Mars_9205733 | GU994211 | 2007 | Human | France | Europe |
| FR_R | KJ873911 | 2013 | Human | Germany | Europe |
| HEV_RKI | FJ956757 | 2005 | Human | Germany | Europe |
| HUN-E67 | EF530662 | 2005 | Human | Hungary | Europe |
| HUN-E132 | FJ641051 | 2008 | Human | Hungary | Europe |
| Donor_2 | KT873490 | 2014 | Human | Ireland | Europe |
| 122.16_S | KC782934 | 2012 | Human | Italy | Europe |
| NL_Patient_106 | KR362850 | 2014 | Human | Netherlands | Europe |
| patZ06 | HM446470 | 2006 | Human | Russia | Europe |
| Nsk2011-1 | JQ065871 | 2011 | Human | Russia | Europe |
| C1 | FJ464745 | 2001 | Human | Spain | Europe |
| F14_3-18 | KP871826 | 2013 | Swine | Estonia | Europe |
| FRHEV4 | JN998606 | 2010 | Polecat | Netherlands | Europe |
| W1-11 | JQ013791 | 2007 | Rabbit | France | Europe |
| NLRA1HEV2013 | KX110047 | 2013 | Rabbit | Netherlands | Europe |
| sw7aAT | HM623775 | 2007 | Swine | Austria | Europe |
| sw11_4bAT | HM623777 | 2008 | Swine | Austria | Europe |
| 1143-09-2 | HQ591362 | 2009 | Swine | Croatia | Europe |
| HEV-516-2 | KU747142 | 2010 | Swine | Denmark | Europe |
| FR-SHEV3e | JQ953665 | 2006 | Swine | France | Europe |
| GiSw | KF303502 | 2006 | Swine | Germany | Europe |
| RP1647 | FJ998010 | 2007 | Swine | Germany | Europe |
| SwHEVEm11IT00 | KJ174068 | 2000 | Swine | Italy | Europe |
| HEV13RS366-35 | KF939864 | 2013 | Swine | Italy | Europe |
| HEV/13RS366-34 | KF939863 | 2012 | Swine | Italy | Europe |
| 1290_2 | KU892219 | 2013 | Swine | Norway | Europe |
| roEF5 | KM058114 | 2009 | Swine | Romania | Europe |
| SI-O11-4 | JF431028 | 2004 | Swine | Slovenia | Europe |
| pig-no.44 | GU953690 | 2010 | Swine | France | Europe |
| Sw serum no.62 B5 Mrs | FJ718764 | 2007 | Swine | France | Europe |
| SwHEVEm73IT00 | KJ174073 | 2000 | Swine | Italy | Europe |
| SwHEV24PGIT13 | KJ508208 | 2013 | Swine | Italy | Europe |
| LODV | KC145142 | 2009 | Swine | Spain | Europe |
| HEV_P006 | KP293758 | 2013 | Swine | United Kingdom | Europe |
| HEV_P427 | KP293770 | 2013 | Swine | United Kingdom | Europe |
| 4 | KR027401 | 2003 | Human | USA | North America |
| 17 | KR027484 | 2004 | Human | USA | North America |
| Kernow-C1 | JQ679014 | 2010 | Human | USA | North America |
| US-C031008 | JN837481 | 2008 | Human | USA | North America |
| Study_ID_3 | KT718044 | 1998 | Human | USA | North America |
| Kernow-C1 | HQ389543 | 2009 | Human | USA | North America |
| Study_ID_1 | KT718045 | 2012 | Human | USA | North America |
| Study_ID_2 | KT718043 | 2002 | Human | USA | North America |
| LBPR-000379 | JN564006 | 2010 | Human | USA | North America |
| 13 | KR027440 | 2004 | Human | USA | North America |
| 16 | KR027473 | 2004 | Human | USA | North America |
| Kernow-C1 | JQ679013 | 2010 | Human | USA | North America |
| CMC-1 | JX565469 | 2010 | Rabbit | USA | North America |
| OD514 | KP255926 | 2012 | Swine | Canada | North America |
| OC585 | KP255934 | 2012 | Swine | Canada | North America |
| A8 | KP255919 | 2011 | Swine | Canada | North America |
| D3-29a | KT778290 | 2014 | Swine | Canada | North America |
| P-C11-1a | KT778284 | 2014 | Swine | Canada | North America |
| OH358 | KP255921 | 2011 | Swine | Canada | North America |
| OA373 | KP255941 | 2012 | Swine | Canada | North America |
| P-D11-1b | KT778276 | 2014 | Swine | Canada | North America |
| OJ168 | KP255946 | 2011 | Swine | Canada | North America |
| OG396 | KP255937 | 2012 | Swine | Canada | North America |
| OE405 | KP255935 | 2012 | Swine | Canada | North America |
| OE416 | KP255948 | 2012 | Swine | Canada | North America |
| OD506 | KP255929 | 2012 | Swine | Canada | North America |
| D10-107a | KT778287 | 2014 | Swine | Canada | North America |
| OC589 | KP255949 | 2012 | Swine | Canada | North America |
| P-D2-4a | KT778280 | 2014 | Swine | Canada | North America |
| P-D2-2c | KT778282 | 2014 | Swine | Canada | North America |
| P-D2-3a | KT778281 | 2014 | Swine | Canada | North America |
| OJ623 | KP255947 | 2012 | Swine | Canada | North America |
| D3-34a | KT778288 | 2014 | Swine | Canada | North America |
| P-D3-2b | KT778278 | 2014 | Swine | Canada | North America |
| OH125 | KP255950 | 2011 | Swine | Canada | North America |
| C11-129a | KT778274 | 2014 | Swine | Canada | North America |
| C11-130a | KT778296 | 2014 | Swine | Canada | North America |
| C11-144a | KT778294 | 2014 | Swine | Canada | North America |
| B285 | KP255939 | 2011 | Swine | Canada | North America |
| G353 | KP255932 | 2011 | Swine | Canada | North America |
| D329 | KP255920 | 2011 | Swine | Canada | North America |
| OH367 | KP255942 | 2011 | Swine | Canada | North America |
| D10-122a | KT778285 | 2014 | Swine | Canada | North America |
| OE406 | KP255936 | 2012 | Swine | Canada | North America |
| OB461 | KP255933 | 2012 | Swine | Canada | North America |
| D2-16b | KT778292 | 2014 | Swine | Canada | North America |
| C186 | KP255930 | 2011 | Swine | Canada | North America |
| OH368 | KP255927 | 2011 | Swine | Canada | North America |
| HB5 | JQ424449 | 2006 | Human | Bolivia | South/Central America |
| HB5BA039 | JQ424448 | 2006 | Human | Bolivia | South/Central America |
| HB5BA038S | JQ424447 | 2006 | Human | Bolivia | South/Central America |
| 49 | KR027788 | 2006 | Human | Brazil | South/Central America |
| Brazilh4.1 | KM502569 | 2009 | Human | Brazil | South/Central America |
| Brazilh4 | KF152884 | 2011 | Human | Brazil | South/Central America |
| Brazilh3 | JX173929 | 2007 | Human | Brazil | South/Central America |
| He_Uy10 | JX880200 | 2010 | Human | Uruguay | South/Central America |
| He_Uy11 | JX880201 | 2010 | Human | Uruguay | South/Central America |
| He_Uy8 | JX880196 | 2010 | Human | Uruguay | South/Central America |
| He_Uy14 | JX880202 | 2010 | Human | Uruguay | South/Central America |
| He_Uy15 | JX880203 | 2010 | Human | Uruguay | South/Central America |
| 4c | KT119523 | 2015 | Human | Costa Rica | South/Central America |
| 2B | KR340542 | 2014 | Human | Costa Rica | South/Central America |
| 2H | KR340547 | 2014 | Human | Costa Rica | South/Central America |
| CUB10D-1999 | EU284749 | 1999 | Human | Cuba | South/Central America |
| CUB2D-2005 | EU284748 | 2005 | Human | Cuba | South/Central America |
| Mex-14 | KX578717 | 1986 | Human | Mexico | South/Central America |
| 2G | KR340546 | 2014 | Human | Costa Rica | South/Central America |
| 2D | KR340544 | 2014 | Human | Costa Rica | South/Central America |
| SA743_SS_VA5 | KU639887 | 2012 | Swine | Colombia | South/Central America |
| CB7 | JQ424440 | 2006 | Swine | Bolivia | South/Central America |
| Swine_037_feces | JN983195 | 2010 | Swine | Brazil | South/Central America |
| BRAsw-117 | JN190069 | 2009 | Swine | Brazil | South/Central America |
| CB2 | JQ424437 | 2006 | Swine | Bolivia | South/Central America |
| CB8 | JQ424441 | 2006 | Swine | Bolivia | South/Central America |
| CB3 | JQ424438 | 2006 | Swine | Bolivia | South/Central America |
| CB5 | JQ424439 | 2006 | Swine | Bolivia | South/Central America |
| EF491206 | EF491206 | 2003 | Swine | Brazil | South/Central America |
| Swine_027_feces | JN983192 | 2010 | Swine | Brazil | South/Central America |
| Swine_027_liver | JN983193 | 2010 | Swine | Brazil | South/Central America |
| BRAsw-13 | JN190065 | 2009 | Swine | Brazil | South/Central America |
| BRAsw-114 | JN190068 | 2009 | Swine | Brazil | South/Central America |
| BRAsw-123 | JN190070 | 2009 | Swine | Brazil | South/Central America |
| BRAsw-112 | JN190067 | 2009 | Swine | Brazil | South/Central America |
| 147 | KP966827 | 2014 | Swine | Brazil | South/Central America |
| 79 | KP966826 | 2014 | Swine | Brazil | South/Central America |
| 49 | KP966825 | 2014 | Swine | Brazil | South/Central America |
| BRAsw-107 | JN190066 | 2009 | Swine | Brazil | South/Central America |
| SW2 | EF591854 | 2009 | Swine | Brazil | South/Central America |
| SW3 | EF591855 | 2009 | Swine | Brazil | South/Central America |
| SW4 | EF591856 | 2009 | Swine | Brazil | South/Central America |
| SA712_SS_OCC5 | KU639883 | 2012 | Swine | Colombia | South/Central America |
| SA740_SS_VA5 | KU639886 | 2012 | Swine | Colombia | South/Central America |
| SD778_SS_VA5 | KU639889 | 2012 | Swine | Colombia | South/Central America |
| SD781_SS_VA4 | KU639890 | 2012 | Swine | Colombia | South/Central America |
| SD786_SS_VA4 | KU639891 | 2012 | Swine | Colombia | South/Central America |
| SD787_SS_VA4 | KU639892 | 2012 | Swine | Colombia | South/Central America |
