# Supplementary material for: Origin and dispersal of Hepatitis E virus
Source: Emerg Microbes Infect. 2018 Feb 7;7:11. doi: 10.1038/s41426-017-0009-6 (PMC5837148; doi:10.1038/s41426-017-0009-6)

Supplementary Figure S1. Phylogeographic analysis of *Orthohepevirus A*. Maximum clade credibility tree for Reg-2. Branches are colored according to inferred ancestral location; posterior support for the location of relevant nodes is shown

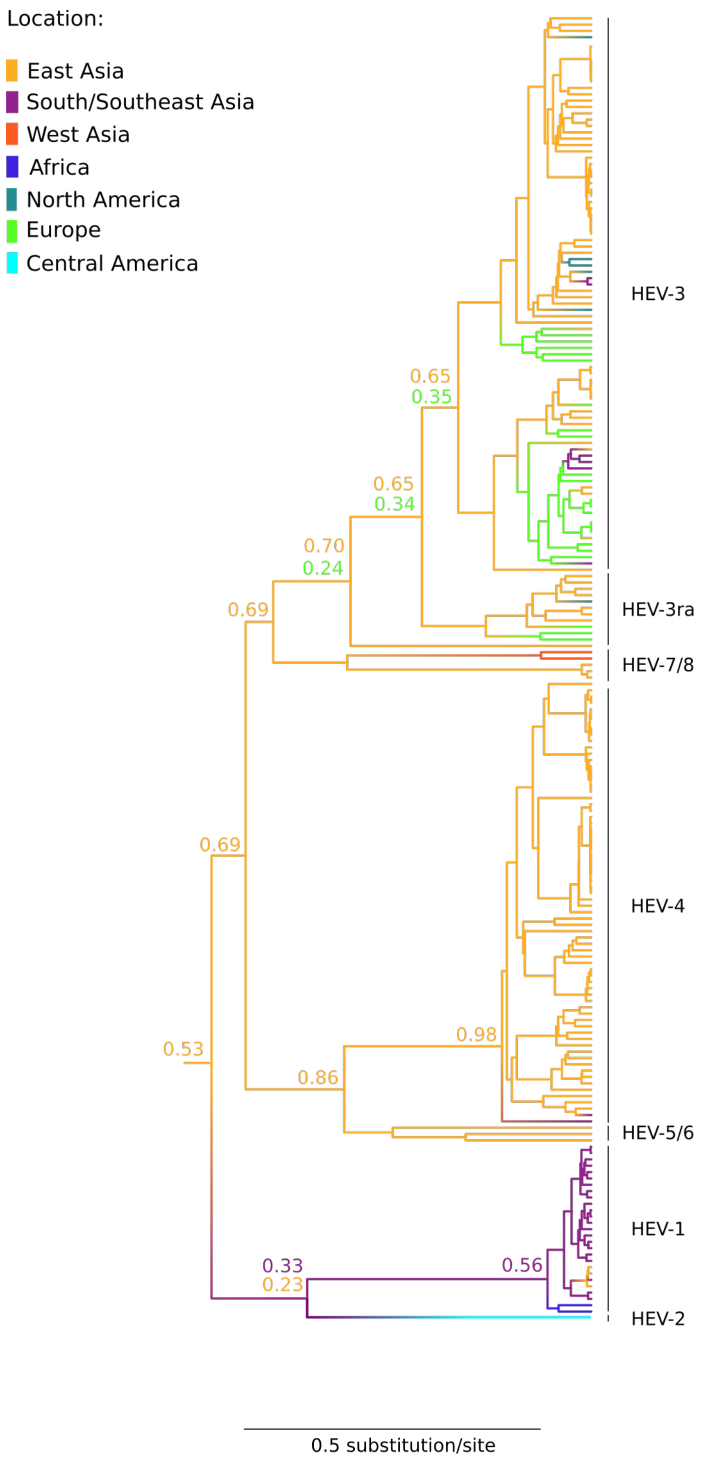

Supplement: Supplementary file 4 — Supplementary Figure S1 [file 41426_2017_9_MOESM4_ESM.pdf]
